# A hybrid framework for improving uncertainty quantification in deep learning-based QSAR regression modeling

**DOI:** 10.1186/s13321-021-00551-x

**Published:** 2021-09-20

**Authors:** Dingyan Wang, Jie Yu, Lifan Chen, Xutong Li, Hualiang Jiang, Kaixian Chen, Mingyue Zheng, Xiaomin Luo

**Affiliations:** 1 Shanghai Key Laboratory of Forensic Medicine, Academy of Forensic Science, Shanghai, 200063 China; 2 University of Chinese Academy of Sciences, No.19A Yuquan Road, Beijing, 100049 China; 3 Drug Discovery and Design Center, State Key Laboratory of Drug Research, Shanghai Institute of Materia Medica, Chinese Academy of Sciences, 555 Zuchongzhi Road, Shanghai, 201203 China

**Keywords:** Uncertainty quantification, Quantitative structure–activity relationship, Bayesian neural network, Applicability domain, Bayesian inference, Error prediction, Artificial intelligence

## Abstract

Reliable uncertainty quantification for statistical models is crucial in various downstream applications, especially for drug design and discovery where mistakes may incur a large amount of cost. This topic has therefore absorbed much attention and a plethora of methods have been proposed over the past years. The approaches that have been reported so far can be mainly categorized into two classes: distance-based approaches and Bayesian approaches. Although these methods have been widely used in many scenarios and shown promising performance with their distinct superiorities, being overconfident on out-of-distribution examples still poses challenges for the deployment of these techniques in real-world applications. In this study we investigated a number of consensus strategies in order to combine both distance-based and Bayesian approaches together with post-hoc calibration for improved uncertainty quantification in QSAR (Quantitative Structure–Activity Relationship) regression modeling. We employed a set of criteria to quantitatively assess the ranking and calibration ability of these models. Experiments based on 24 bioactivity datasets were designed to make critical comparison between the model we proposed and other well-studied baseline models. Our findings indicate that the hybrid framework proposed by us can robustly enhance the model ability of ranking absolute errors. Together with post-hoc calibration on the validation set, we show that well-calibrated uncertainty quantification results can be obtained in domain shift settings. The complementarity between different methods is also conceptually analyzed.

**Supplementary Information:**

The online version contains supplementary material available at 10.1186/s13321-021-00551-x.

## Introduction

With the increasing scale of available datasets, deep learning methods have made tremendous impact in the chemical domain [1]. However, most works in this area have focused on improving model accuracy, less attention has been paid for quantifying the uncertainty of predictions given by the model. Uncertainty quantification refers to estimating the confidence level of a model output. Reliable estimation of this certainty is often crucial for high-stacks problems, especially drug design and discovery [2–4]. For example, in the scenario of virtual screening, molecules with high predictive activity are chosen for further experimental verification. Through this process it is always expected that the molecules with unreliable predictions can be excluded in order to avoid wasting time and money [5]. However, a deterministic model cannot give such information. This example shows that numerical results without a measure of veracity do not contain enough information for decision making [6].

Given the importance of uncertainty quantification, a plethora of methods have been proposed so far and employed in various cheminformatics tasks such as molecular property prediction [7], chemical reaction prediction [8], material property prediction [9], NMR spectral property prediction [10] and interatomic potential prediction [11]. Broadly speaking, current mainstream uncertainty quantification methods used in the chemical domain can be divided into two categories: distance-based approaches and Bayesian approaches.

The core of distance-based approaches is the traditional concept of applicability domain (AD). AD estimates the chemical space in which the model could give reliable predictions. It is generally accepted that if a test sample is too remote from the training set, its prediction is likely to be unreliable. While the common goal is the same, the representation of the distance between a molecule and the model training set is varied across different distance-based methods. Many classical methods use feature space distance defined by molecular fingerprints [12–17], while some recent studies have shown that the distance in latent space may yield superior performance [18, 19].

Bayesian approaches encompass a diverse group of strategies with strong theoretical guarantee which enjoyed a recent reconstruction as a result of the improvement of computing power [20–22]. The underlying assumption behind the Bayesian approach is that the model weights and predictions are no longer definite point estimates but probability distributions which allows uncertainties of predictions to be taken into the model naturally [23]. By fitting a defined model to the observed data (training set), the posterior distribution of model weights can be theoretically obtained and used to make inference. The total uncertainty of a prediction is then quantified as its posterior variance. Interestingly, in Bayesian modelling, the total uncertainty can be decomposed into two components: aleatoric uncertainty which captures the noise of labels and epistemic uncertainty which results from the lack of training data [7, 24]. Many researches have made use of this desired property to identify the main source of uncertainty for their specific tasks [9, 25].

Despite the progress mentioned above, the reliability and applicable conditions of both distance-based and Bayesian methods are still limited by some challenges. On the one hand, the measure of chemical space distance is ambiguous and the threshold for classifying reliable predictions is hard to define. Also, the distance-to-model metric lacks the information of stochasticity arising from the data. On the other hand, although the computational intractability of Bayesian methods has been eased by several approximating ways [26], Bayesian approaches are reported to tend to make overconfident predictions for out-of-domain examples [27, 28]. In this context, we make the assumption that combining both distance-based and Bayesian methods represents a feasible strategy which can minimize the intrinsic drawbacks of these methods.

The complementarity between Bayesian methods and distance-based methods can be viewed in a more theoretic way. Some recent studies proposed that except for the epistemic uncertainty and aleatoric uncertainty that have been included in the Bayesian approach, distributional uncertainty is another source of uncertainty that needs to be considered [29]. Distributional uncertainty describes that the model is unfamiliar with the test data and thus cannot confidently make predictions, no matter for the label or the data noise. Some uncertainty quantification methods can explicitly model distributional uncertainties, but at the same time need out-of-domain samples during training time, which is unrealistic in real-word applications [30]. To this end, distance-based methods here play a similar role as distributional uncertainty modeling methods to estimate whether a sample is out-of-domain, which makes up the shortcomings of Bayesian methods.

In this study, we investigated the performance of several consensus strategies that combine both distance-based and Bayesian uncertainty quantification approaches in the context of deep learning-based QSAR regression modeling. The value of performing post-hoc calibration on a leave-out validation set was also studied. Special emphasis was put on model’s ability of ranking absolute errors and providing calibrated uncertainty quantification results. The performance of different models was benchmarked on 24 biological regression datasets. We found that the consensus model showed improved performance over individual methods in both in-domain and out-of-domain settings.

## Methods and datasets

### Problem definition

Suppose we are given a training set with $m$ samples ${\mathcal{D}}^{A} = \left( {\left\{ {x_{i}^{A} } \right\}_{i = 1}^{m} ,\left\{ {y_{i}^{A} } \right\}_{i = 1}^{m} } \right) = \left( {{\varvec{X}}^{A} ,{\varvec{Y}}^{A} } \right)$, a validation set with $n$ samples ${\mathcal{D}}^{B} = \left( {\left\{ {x_{i}^{B} } \right\}_{i = 1}^{n} ,\left\{ {y_{i}^{B} } \right\}_{i = 1}^{n} } \right) = \left( {{\varvec{X}}^{B} ,{\varvec{Y}}^{B} } \right)$ and a test set with $l$ samples ${\mathcal{D}}^{C} = \left( {\left\{ {x_{i}^{C} } \right\}_{i = 1}^{l} ,\left\{ {y_{i}^{C} } \right\}_{i = 1}^{l} } \right) = \left( {{\varvec{X}}^{C} ,{\varvec{Y}}^{C} } \right)$. Here $x$ represents an input molecule and $y \in {\mathbb{R}}$ is a real-valued property. A deep learning-based regression model ${\mathcal{M}}$ parameterized by weights ${\varvec{\theta}}$ is trained on ${\mathcal{D}}^{A}$ with early stopping on ${\mathcal{D}}^{B}$. ${\mathcal{M}}$ is then used to make predictions on ${\mathcal{D}}^{B}$ and ${\mathcal{D}}^{C}$ which can be represented by vectors $\hat{\user2{Y}}^{B} = \left\{ {\hat{y}_{i}^{B} } \right\}_{i = 1}^{n}$ and $\hat{\user2{Y}}^{C} = \left\{ {\hat{y}_{i}^{C} } \right\}_{i = 1}^{l}$. Signed error vectors are defined by ${\varvec{E}}^{B} = \left\{ {\hat{y}_{i}^{B} - y_{i}^{B} } \right\}_{i = 1}^{n} = \left\{ {e_{i}^{B} } \right\}_{i = 1}^{n}$ and ${\varvec{E}}^{C} = \left\{ {\hat{y}_{i}^{C} - y_{i}^{C} } \right\}_{i = 1}^{l} = \left\{ {e_{i}^{C} } \right\}_{i = 1}^{l}$. It can be assumed that [31] the error of each prediction $e_{i}^{C}$ is a random variable following a Gaussian distribution with a zero mean and a specific variance $\left( {\sigma_{i}^{C} } \right)^{2}$:

1 $$\begin{array}{*{20}c} {e_{i}^{C} \sim N\left( {0,\left( {\sigma_{i}^{C} } \right)^{2} } \right)} \\ \end{array}$$

where $\left( {\sigma_{i}^{C} } \right)^{2}$ is a function of the trained model and $x_{i}^{C}$. Given ${\mathcal{D}}^{A}$, ${\mathcal{D}}^{B}$, ${\mathcal{M}}$ and $x_{i}^{C}$, an uncertainty quantification method $Q$ is used to give a relative or direct estimation $Q\left( {x_{i}^{C} ;{\mathcal{D}}^{A} , {\mathcal{D}}^{B} ,{\mathcal{M}}} \right)$ for $\left( {\sigma_{i}^{C} } \right)^{2}$. When it is clear from the context, this estimation will be directly referred to as $Q\left( {x_{i}^{C} } \right)$. Suppose there are $t$ individual uncertainty quantification methods $Q_{1} , \ldots ,Q_{t}$, each of which could give a set of uncertainty estimation on ${\mathcal{D}}^{C}$ named as ${\varvec{U}}_{j}^{C} = \left\{ {Q_{j} \left( {x_{i}^{C} } \right)} \right\}_{i = 1}^{l} , j = 1, \ldots ,t.$ We make the assumption that ${\varvec{U}}_{1}^{C} , \ldots$,${\varvec{U}}_{t}^{C}$ can be combined through a consensus model $f$ into a stronger estimation ${\varvec{U}}_{*}^{C} = f\left( {{\varvec{U}}_{1}^{C} , \ldots {\varvec{U}}_{t}^{C} } \right) = \left\{ {Q_{*} \left( {x_{i}^{C} } \right)} \right\}_{i = 1}^{l}$.

### Datasets

24 bioactivity datasets gathered by Cortés-Ciriano et al. were used to benchmark the performance of different uncertainty quantification methods in this study [32]. Each dataset consists of bioactive small molecules for a specific drug target and IC50 values extracted from ChEMBL [33]. The number of data points per target varies from 203 (A2a) to 5207 (HERG). When multiple pIC50 values were available for the same compound, the average pIC50 value was calculated and taken as the label. Further information and all datasets can be found in ref [32, 34–36].

### Graph convolutional neural network (GCNN)

Directed Message Passing Neural Network (D-MPNN) [37] was used in this study to conduct molecular activity prediction. D-MPNN is a kind of graph convolutional neural networks which uses messages centered on directed bonds instead of atoms to avoid unnecessary message-passing loop. Unless otherwise noted, the default hyperparameter setting from the Chemprop package [37] was used to train the model. Noam learning rate schedule [38] was adopted to dynamically adjust learning rate. Early stopping was used to avoid over-fitting during training. At the end of each epoch, the loss on the validation set was calculated and recorded. If the loss did not decrease over 50 epochs, the training process was stopped and the best model on the validation set was taken as the final model for evaluation. Mean–variance loss was used as the loss function to estimate aleatoric uncertainty, for more details see the next subsection.

### Mean-variance estimation (MVE)

The basic assumption of MVE is that the labels of a regression dataset are normal variables with different variances which arise from the experimental noise [27]. This is also called aleatoric uncertainty in Bayesian uncertainty analysis. MVE gives the estimation of the mean and variance for each data point with maximum likelihood estimation (MLE). In this way, the prediction of the model is a distribution rather than a single point. Practically, the output of GCNN is branched into two predictions: the mean $\mu \left( {{\varvec{\theta}},x} \right)$ and the variance $v\left( {{\varvec{\theta}},x} \right)$. $v\left( {{\varvec{\theta}},x} \right)$ is taken as the result of MVE as the estimated uncertainty during the prediction process. A loss function proportional to the negative log-likelihood of the normal distribution instead of the traditional mean squared error (MSE) loss is used to optimize the weights ${\varvec{\theta}}$:

2 $$\begin{array}{*{20}c} {L\left( \user2{\theta } \right) = \frac{1}{N}\mathop \sum \limits_{{i = 1}}^{N} \left( {\frac{{\left( {y_{i} - \mu \left( {\user2{\theta },x_{i} } \right)} \right)^{2} }}{{2v\left( {\user2{\theta },x_{i} } \right)}} + \frac{1}{2}\ln v\left( {\user2{\theta },x_{i} } \right)} \right)} \\ \end{array}$$

The output $v\left( {{\varvec{\theta}},x} \right)$ of a model trained in this way is taken as the estimated aleatoric uncertainty $Q_{A} \left( x \right)$:

3 $$\begin{array}{*{20}c} {Q_{A} \left( {x_{i}^{C} } \right) = v\left( {{\varvec{\theta}},x_{i}^{C} } \right)} \\ \end{array}$$

### Ensemble (ENS)

Except for label noise inherent in dataset, the randomness of training process is another source of uncertainty. With the same loss on the training set, there are multiple possible model weights that are able to explain the data. This is called epistemic uncertainty in Bayesian uncertainty estimation, which results from the lack of knowledge at certain regions of the feature space, and can be neutralized by increasing training data in those low-density regions. In this study, an ensemble approach was used to obtain this kind of uncertainty. For each training process, a set of models $\left\{ {{\mathcal{M}}_{k} } \right\}_{k = 1}^{K}$ were trained parallelly with different random initial weights. The ensemble variance defined as the following equations is then used to estimate the epistemic uncertainty $Q_{E} \left( {x_{i}^{C} } \right)$ for the test sample $x_{i}^{C}$:

4 $$\begin{array}{*{20}c} {{\tilde{\mathcal{M}}}\left( {x_{i}^{C} } \right) = \frac{1}{K}\mathop \sum \limits_{k = 1}^{K} {\mathcal{M}}_{k} \left( {x_{i}^{C} } \right)} \\ \end{array}$$

5 $$\begin{array}{*{20}c} {Q_{E} \left( {x_{i}^{C} } \right) = \frac{1}{K}\mathop \sum \limits_{k = 1}^{K} \left( {{\tilde{\mathcal{M}}}\left( {x_{i}^{C} } \right) - {\mathcal{M}}_{k} \left( {x_{i}^{C} } \right)} \right)^{2} } \\ \end{array}$$

where ${\mathcal{M}}_{k} \left( {x_{i}^{C} } \right)$ is the prediction of the *k*-th model, ${\tilde{\mathcal{M}}}\left( {x_{i}^{C} } \right)$ is the average prediction of ensemble models and $Q_{E} \left( {x_{i}^{C} } \right)$ is the estimation of the uncertainty given by the ensemble variance. If MVE is used during training (as shown in Eq. 2), the predicted mean value $\mu \left( {{\varvec{\theta}}_{k} ,x_{i}^{C} } \right)$ is taken as ${\mathcal{M}}_{k} \left( {x_{i}^{C} } \right).$ This approach was first proposed as Deep Ensemble [39] and has been widely used in other researches. Although there still exist other popular ensemble techniques for obtaining ensemble models, such as Monte Carlo dropout [21, 40], hyperparameter ensemble [41] and bootstrapping [42], a recent study has shown that many of them are essentially equivalent to simply ensemble of several independently trained networks as Deep Ensemble does [43], which has become a practical alternative to improve the accuracy of deep learning models. It has also been reported that Deep Ensemble significantly outperforms other ensemble techniques on a variety of machine learning tasks [27, 28]. Another advantage for Deep Ensemble is that it is based on the standard training pipeline without additional computation burden, which makes the implementation much simpler.

### Feature space distance (FDIST)

The feature space distance between the test sample and the training dataset has long been used to define the AD for machine learning models. A variety of approaches have been proposed in the literature over the years for measuring the feature space distance [13–15, 17, 44]. Considering the generality of the method, a rather simple but robust measurement named SIMILARITYNEARIST1 [12, 14] is used in this study. Here, each molecule is represented by the MinHash fingerprint6 (MHFP6) [45], which removes the curse of dimensionality and outperforms ECFP4 [46] in analog recovery experiments. The uncertainty of a test sample is given by the Tanimoto distance [47] of that with the nearest sample in the training set.

6 $$\begin{array}{*{20}c} {Q_{F} \left( {x_{i}^{C} } \right) = min\left\{ {FDIST\left( {x_{i}^{C} , x_{j}^{A} } \right)} \right\}_{j = 1}^{m} } \\ \end{array}$$

### Latent space distance (LDIST)

GCNN is able to build a learned molecular representation and further engineer it automatically to amplify the effects of task-related features and limit the effects of weakly-informative features. When the model could extract the task-related features of molecules successfully, the distance in latent space is extremely meaningful to stand for the similarity of two molecules on a specific task. As a result, latent space distance was recently proposed as a novel and efficient strategy of uncertainty quantification [19]. Here, the cosine distance in the final layer latent space was used to assess the similarity of a test point and the training dataset. The number of nearest neighbors was also set to one as used in the calculation of FDIST.

7 $$\begin{array}{*{20}c} {Q_{L} \left( {x_{i}^{C} } \right) = min\left\{ {LDIST\left( {x_{i}^{C} , x_{j}^{A} } \right)} \right\}_{j = 1}^{m} } \\ \end{array}$$

**Fig. 1:**
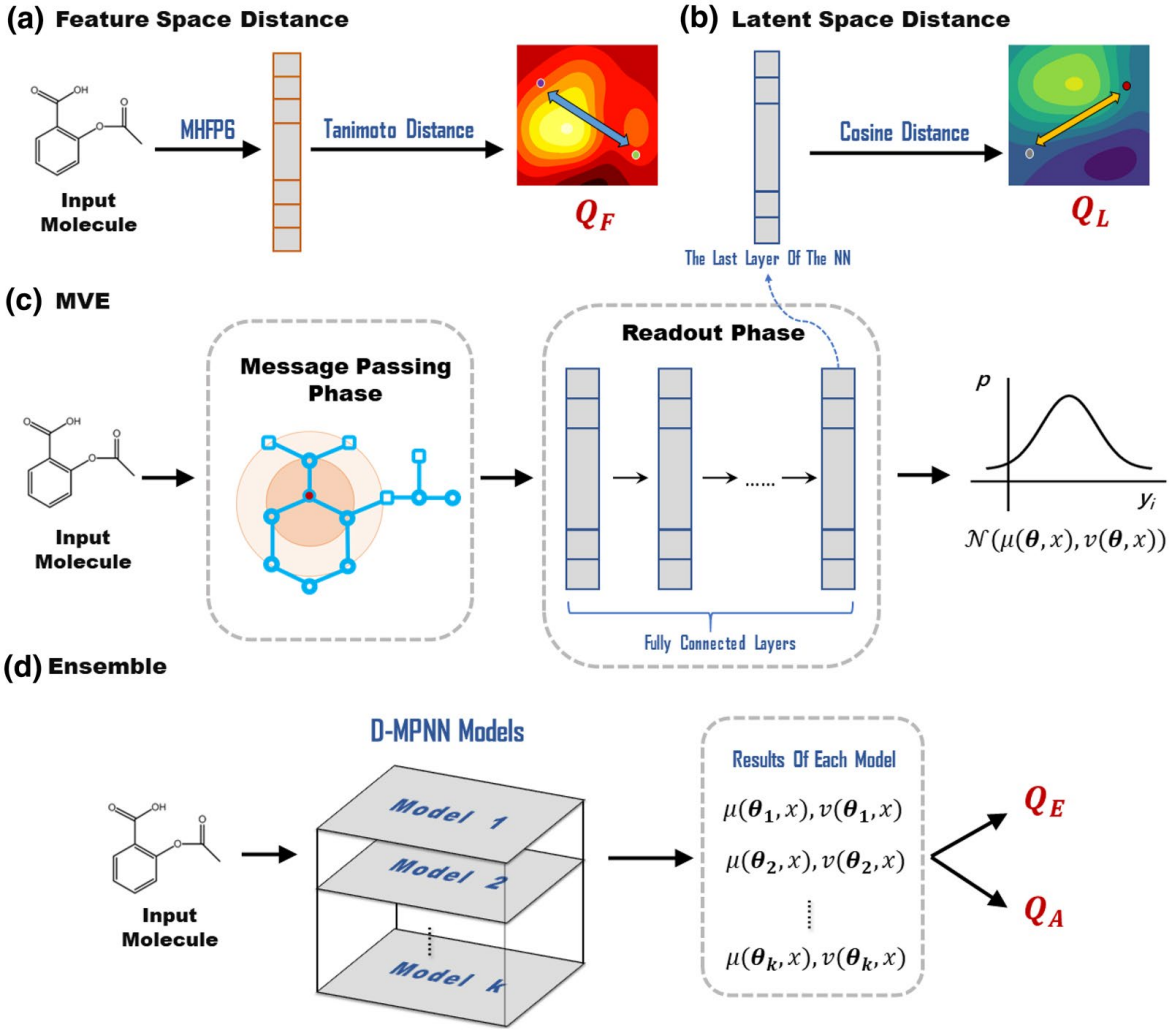
Illustration of each individual model. **a** Feature space distance (FDIST); **b** Latent space distance (LDIST); **c** Mean–variance estimation (MVE); **d** Ensemble (ENS)

In practice, 10 models were trained with randomly initialized weights for a given training/validation/testing split. The arithmetic mean of these 10 predictions was taken as the ensemble predictions on the validation set and the test set as shown in Eq. 4, while the variance of which was taken as the result of ENS as shown in Eq. 5. Noticed that FDIST is independent with the model weights, but the results of MVE and LDIST vary as the weights change. In this case the results of these two approaches were also averaged over the 10 models as is done for the predictions. A graph illustration of each individual method is shown in Fig. 1.

### Bayesian approach (BYS)

With the MVE approach, the prediction of a model is not a definite number but a random variable. Given a model with weights ${\varvec{\theta}}$ and input $x$, the conditional expectation and variance of the prediction $\hat{y}$ are:

8 $$\begin{array}{*{20}c} {E\left[ {\hat{y}{|}{\varvec{\theta}},x} \right] = \mu \left( {{\varvec{\theta}},x} \right)} \\ \end{array}$$

9 $$\begin{array}{*{20}c} {var\left( {\hat{y}{|}{\varvec{\theta}},x} \right) = v\left( {{\varvec{\theta}},x} \right)} \\ \end{array}$$

where $\mu \left( \cdot \right)$ and $v\left( \cdot \right)$ are two deterministic functions. Under the Bayesian perspective, the weights of a trained model are also random variables following posterior distribution $p\left( {{\varvec{\theta}}{|}{\mathcal{D}}^{A} } \right)$:

10 $$\begin{array}{*{20}c} {p\left( {{\varvec{\theta}}{|}{\mathcal{D}}^{A} } \right) = \frac{{p\left( {\varvec{\theta}} \right)p\left( {{\mathcal{D}}^{A} {|}{\varvec{\theta}}} \right)}}{{\smallint p\left( {\varvec{\theta}} \right)p\left( {{\mathcal{D}}^{A} {|}{\varvec{\theta}}} \right)d{\varvec{\theta}}}}} \\ \end{array}$$

where $p\left( {\varvec{\theta}} \right)$ is the prior and $p\left( {{\mathcal{D}}^{A} {|}{\varvec{\theta}}} \right)$ is the likelihood. Combining Eqs. 8–10, the posterior distribution of the prediction is:

11 $$\begin{array}{*{20}c} {p\left( {\hat{y}{|}x,{\mathcal{D}}^{A} } \right) = \smallint p\left( {\hat{y}{|}{\varvec{\theta}},x} \right)p\left( {{\varvec{\theta}}{|}{\mathcal{D}}^{A} } \right)d{\varvec{\theta}}} \\ \end{array}$$

whose expectation and variance are:

12 $$\begin{array}{*{20}c} {E\left[ {\hat{y}{|}x,{\mathcal{D}}^{A} } \right] = E\left[ {\mu \left( {{\varvec{\theta}},x} \right)} \right], {\varvec{\theta}} \sim p\left( {{\varvec{\theta}}{|}{\mathcal{D}}^{A} } \right)} \\ \end{array}$$

13 $$\begin{array}{*{20}c} {var\left( {\hat{y}{|}x,{\mathcal{D}}^{A} } \right) = E\left[ {v\left( {{\varvec{\theta}},x} \right)} \right] + var\left( {\mu \left( {{\varvec{\theta}},x} \right)} \right), {\varvec{\theta}} \sim p\left( {{\varvec{\theta}}{|}{\mathcal{D}}^{A} } \right)} \\ \end{array}$$

Equation 13 shows how to define and calculate uncertainty of model predictions in a Bayesian way. It is noticed that according to Eq. 13, the total uncertainty $var\left( {\hat{y}{|}x,{\mathcal{D}}^{A} } \right)$ is decomposed into two components, aleatoric uncertainty (the former one) and epistemic uncertainty (the latter one). Due to the large number of parameters contained in ${\varvec{\theta}}$ which leads to high dimensional integrals, calculation of Eq. 13 is intractable. Many methods have focused on finding strategies to make efficient approximation. As a realization of this framework, Scalia et al. proposed that the outputs of MVE and ENS can be added to obtain an approximation of Bayesian total uncertainty (as shown in Eq. 14), leading to a better performance compared with using single method alone [27]. This method which can also be treated as a simple aggregation strategy was used as a baseline model in this study for comparison and referred to as BYS.

14 $$\begin{array}{*{20}c} {Q_{BYS} \left( {x_{i}^{C} } \right) = var\left( {\hat{y}{|}x_{i}^{C} ,{\mathcal{D}}^{A} } \right) \approx Q_{A} \left( {x_{i}^{C} } \right) + Q_{E} \left( {x_{i}^{C} } \right)} \\ \end{array}$$

### Consensus methods and corresponding evaluation metrics

To combine the outputs of single models $\left\{ {{\varvec{U}}_{i}^{C} } \right\}_{i = 1}^{t}$, two weighted consensus strategies were considered in this study: (1) weighted averaging focusing on improving the ranking ability; and (2) NLL (negative log-likelihood) calibration focusing on improving the calibration ability, both of which take the form of a linear combination of predictions provided by individual models to get the final uncertainty estimation, as follows:

15 $$\begin{array}{*{20}c} {{\varvec{U}}_{*}^{C} = \mathop \sum \limits_{i = 1}^{t} \left( {w_{0} + w_{i} {\mathbb{H}}({\varvec{U}}_{i}^{C} }) \right)} \\ \end{array}$$

where ${\varvec{W}} = \left\{ {w_{i} } \right\}_{i = 1}^{t}$ are the positive weighting coefficients, $w_{0}$ is the bias, $\left\{ {{\varvec{U}}_{i}^{C} } \right\}_{i = 1}^{t}$ are denormalized outputs of individual models, ${\mathbb{H}}\left( \cdot \right)$ is a normalization function and ${\varvec{U}}_{*}^{C}$ is the final outputs. Next, we will explain the meaning of ranking and calibration ability and how we obtain these abilities with corresponding methods.

### Weighted averaging

In general, an ideal uncertainty quantification method should assign higher uncertainty values to predictions with higher absolute errors. We name this as the ranking ability of the model. Assuming that each individual model has already possessed ranking ability to some degree, the problem now is how to combine these models into a stronger one. A weighted averaging approach as shown in Eq. 15 is used to achieve this goal. However, directly adding the results of different individual models is not applicable owing to different quantities and units of individual predictions. In this context, a normalization is performed for each model’s results at first. Two classical normalization strategies, z-score normalization (referred to as *Zscore*) and min–max normalization (referred to as *MinMax*), were tested in this study. We also considered to directly transform the results into rankings (referred to as *Rank*) before performing weighted averaging.

To determine the weights ${\varvec{W}}$ in Eq. 15, without any prior knowledge, the most intuitive way is to use arithmetic mean (referred to as *Unweighted*), where $w_{0} = 0$ and $w_{1} = \ldots = w_{t} = 1$. We also explored the possibility of assigning higher weight for the method showing better performance on the validation set (referred to as *Weighted*). Since the ranking ability can be quantitatively measured by the Spearman Correlation Coefficient (SCC) between the predictions and absolute errors, Eq. 16 is used to determine the weight for each individual model.

16 $$\begin{array}{*{20}c} {w_{i} = \max \left( {0, \,SCC\left( {{\varvec{U}}_{i}^{B} , \left| {{\varvec{E}}^{B} } \right|} \right)} \right), \quad i = 1, \ldots ,t} \\ \end{array}$$

Practically we found that SCCs can rarely simultaneously be negative for all individual models on the validation set. In this case the arithmetic mean would be used instead.

Confidence curve is a usual way to visually assess the ranking ability of the model. To draw this curve, the most uncertain samples are successively removed and the mean absolute error (MAE) is calculated for the remaining predictions. The confidence curve is plotted by showing how the value of MAE varies as the function of confidence percentile. A monotonic decreasing confidence curve would be expected for an ideal uncertainty estimator. When two curves were compared in parallel, the one with a smaller AUC (area under the curve) should be regarded as the better one. SCC between the estimated uncertainties and the absolute errors on the test set was used to quantitatively measure the ranking ability, following the work of Hirschfeld et al. [48].

### NLL calibration (NLLCAL)

Even if a consensus model shows a perfect ranking ability, which we would not expect since $e_{i}^{C}$ is considered to be a random variable sampled from a zero-mean normal distribution as shown in Eq. 1, it still remains unknown whether $Q_{*} \left( {x_{i}^{C} } \right)$ equals to $\left( {\sigma_{i}^{C} } \right)^{2}$ in value. The weighted averaging methods described in the previous subsection are only aimed at giving estimation of rankings for absolute errors instead of directly predicting the uncertainty $\left( {\sigma_{i}^{C} } \right)^{2}$. To do this, a post-hoc calibration on the validation set with MLE strategy is used. In MLE, we hope to learn a set of weights ${\varvec{W}}$ that maximize the likelihood of observing errors on the validation set ${\varvec{E}}^{B}$, given by $\mathop \prod \limits_{i = 1}^{n} p( {e_{i}^{B} {|}( {\sigma_{i}^{B} } )^{2} = Q_{*} ( {x_{i}^{B} } )} )$. This is achieved by minimizing the following NLL loss function:

17 $$\begin{array}{*{20}c} {L\left( {\varvec{W}} \right) = \mathop \sum \limits_{i = 1}^{n} \left( {\ln \left( {Q_{*} \left( {x_{i}^{B} } \right)} \right) + \frac{{\left( {e_{i}^{B} } \right)^{2} }}{{Q_{*} \left( {x_{i}^{B} } \right)}}} \right)} \\ \end{array}$$

where $Q_{*} \left( {x_{i}^{B} } \right)$ is the *i*-th element of ${\varvec{U}}_{*}^{B} = \mathop \sum \limits_{j = 1}^{t} \left( {w_{0} + w_{j} {\varvec{U}}_{j}^{B} } \right)$. This method will be referred to as *NLLCAL* from now on. A similar MLE-based calibration approach is adopted by Janet et al. in which only latent distance information is used [19]. The idea of fitting absolute errors against several uncertainty metrics on the validation set to construct a hybrid error prediction model has also been explored by a set of work of Sheridan in the context of machine-learning-based QSAR modeling earlier [14, 49, 50].

Whether $Q_{*} \left( {x_{i}^{C} } \right)$ is equivalent to $\left( {\sigma_{i}^{C} } \right)^{2}$ can be evaluated under error-based view or confidence-based view [27, 48]. Error-based calibration means that given the predicted mean $\hat{y}$ and uncertainty $Q\left( x \right)$, the following condition is met:

18 $$\begin{array}{*{20}c} {{\mathbb{E}}_{{\left( {x,y} \right)\sim {\mathcal{D}}^{C} }} \left[ {\left( {\hat{y} - y} \right)^{2} {|}Q\left( x \right) = \sigma^{2} } \right] = \sigma^{2} } \\ \end{array}$$

To assess error-based calibration, we followed the method proposed by Levi et al. [51]. According to this procedure, the test set is divided into multiple bins according to the predicted uncertainties ${\varvec{U}}_{*}^{C}$. In this study, each bin is set to have 20 samples with close predicted uncertainty. A scatter plotting between the root mean squared error (RMSE) and the root of average predicted uncertainty of each bin is then drawn. For a perfect calibrated model, points should be distributed around the diagonal line of the plotting. Expected normalized calibration error (ENCE) is defined to quantitatively measure the error-based calibration error:

19 $$\begin{array}{*{20}c} {ENCE = \frac{1}{N}\mathop \sum \limits_{i = 1}^{N} \frac{{\left| {\sqrt {mVAR\left( i \right)} - \sqrt {MSE\left( i \right)} } \right|}}{{\sqrt {mVAR\left( i \right)} }}} \\ \end{array}$$

where $N$ equals to the number of bins, $mVAR\left( i \right)$ is the average predicted uncertainty over the *i*-th bin and $MSE\left( i \right)$ is the MSE over the *i*-th bin.

Obviously, there is no need to make any distribution assumption of errors for checking whether a model is error-based calibrated. However, if we want to define the confidence interval of error with a specified confidence level, such as 80%, for each test sample using the uncertainty value obtained, a zero-mean Gaussian distribution assumption is usually required, as shown in Eq. 1. Under the Gaussian distribution assumption, accuracy of the confidence interval is a function of confidence level ranging from 0 to 100%. According to this, the reliability diagrams can be plotted to check whether a model is confidence-based calibrated. Here the accuracy of a confidence interval is defined as the proportion of errors falling into the interval. A perfectly confidence-based calibrated model should obtain a diagonal line for the reliability diagram. In other words, for a *x*% confidence interval, it should always be observed that *x*% errors fall into it. Expected calibration error (ECE) is used to give a scalar statistic of miscalibration rate, defined as:

20 $$\begin{array}{*{20}c} {ECE = \frac{1}{100}\mathop \sum \limits_{i = 1}^{100} \left| {acc\left( {i\% } \right) - i/100} \right|} \\ \end{array}$$

where $acc\left( {i\% } \right)$ refers to the accuracy of *i*% confidence interval.

### Splitting strategy

For each target we followed the standard fivefold cross validation (fivefold CV) protocol as is commonly done in the development pipeline of QSAR models. To analyze the performance of the model in different application scenarios, three different splitting strategies were adopted. Each strategy performed a fivefold CV split on the full dataset for each target, but with varying degrees of domain shift. For each split, the ratio of training/validation/testing is always 60/20/20. We named these three splitting strategies as IVIT, IVOT and OVOT, where V refers to “validation set”, T refers to “test set”, I refers to “in-domain” and O refers to “out-of-domain”. The difference between them will be briefly discussed next.

IVIT (in-domain validation set; in-domain test set) is just the standard random splitting strategy making the training, validation, and testing sets cover the same chemical space. This strategy is widely used, but it is reported that random-split validation is tended to give over-optimistic results [1]. Accordingly, a stricter splitting method, cluster cross-validation [52], which can guarantee that compounds of different clusters cover disparate chemical spaces was adopted in this study.

We used the single-linkage algorithm to carry out the fivefold cluster CV. Single-linkage algorithm is a type of hierarchical clustering depending on the smallest dissimilarity between two examples $\left( {x_{i} ,\, x_{j} } \right)$ respectively from two different clusters $\left( {C_{i} , \,C_{j} } \right)$, which can guarantee that the minimum distance between any two folds is larger than a given threshold, as the following equation shows:

21 $$\begin{array}{*{20}c} {D\left( {C_{i} ,\,C_{j} } \right) = \mathop {\min }\limits_{{x_{i} \in C_{i} ,\,x_{j} \in C_{j} }} dist\left( {x_{i} ,x_{j} } \right) > d_{cutoff} } \\ \end{array}$$

where $dist\left( {\cdot,\cdot} \right)$ is the distance function and $d_{cutoff}$ is the threshold used. In this study, $dist$ was defined by the Tanimoto distance on binarized ECFP4 fingerprints [46], and $d_{cutoff}$ was set to 0.3. Under this configuration, for example, the compounds in HERG were clustered into 1690 clusters with 131 compounds for the largest cluster, and the compounds in JAK2 were clustered into 534 clusters with 324 compounds for the largest cluster. These clusters were then united into five different folds with approximately the same size. For OVOT (out-of-domain validation set; out-of-domain test set), two folds were selected as the validation set and the test set, respectively, and the remaining three folds as the training set. For IVOT (in-domain validation set; out-of-domain test set), one fold was selected as the test set at each time and the remaining four folds were further mixed and randomly split into the training set and the validation set in a ratio of 60:20.

OVOT has been adopted by some previous studies to evaluate the performance of uncertainty quantification models in an out-of-domain setting [48]. However, IVOT is a better way to simulate real usage scenarios since typically we would not intentionally use an out-of-domain validation set for early stopping. What’s more, in this study the validation set functions as a calibration set for weighted averaging and NLLCAL. IVOT provides a better way of checking whether these methods are robust to dataset shift [28].

## Results and discussion

### Motivation of building hybrid uncertainty quantification model

First of all, we would like to discuss the motivation of incorporating the distance information into traditional Bayesian framework according to the equations given in the “Methods and datasets” section. As shown in Eq. 13, the definition of Bayesian uncertainty is the posterior variance $var(\hat{y}|x_{i}^{C} ,\,D^{A} )$, which can be further approximated by adding $Q_{A}$ and $Q_{E}$ together. However, we suppose that there exists a discrepancy between the Bayesian uncertainty $var(\hat{y}|x_{i}^{C} ,\,D^{A} )$ and the absolute error $\left| {e_{i}^{C} } \right|$ which is actually the goal we concern about. A low $var(\hat{y}|x_{i}^{C} ,D^{A} )$ does not guarantee that $\hat{y}_{i}^{C}$ is close to the label $y_{i}^{C}$. That is to say, a model may be quite “certain” but the prediction is “wrong”. This is common for many out-of-domain samples, for which it has been observed that Bayesian approaches usually give overconfident estimation. A typical scenario is that the training set is biased, in which the positive samples contain low dimensional properties, for example, molecular weights, that can be used to easily identify positives and negatives. Considering an extreme case where nearly all positive molecules possess biphosphate groups while the negative ones do not (in fact this is a real case for target FPPS in DUD-E [53]). During training it is very likely that the neural network maps all molecules with biphosphate groups into a neighboring region in the latent distance. For an out-of-domain molecule (defined on the chemical space) containing a biphosphate group, $var(\mu ({\varvec{\theta}},\,x))$ will be quite small since the model takes it as an in-domain sample (defined on the latent space) that can be well explained by the posterior weights. On the other hand, the FDIST will not be affected by the biased data and give important prior knowledge that the prediction is rather uncertain owing to the dissimilar molecular structure.

### Consensus models outperform individual models

**Fig. 2:**
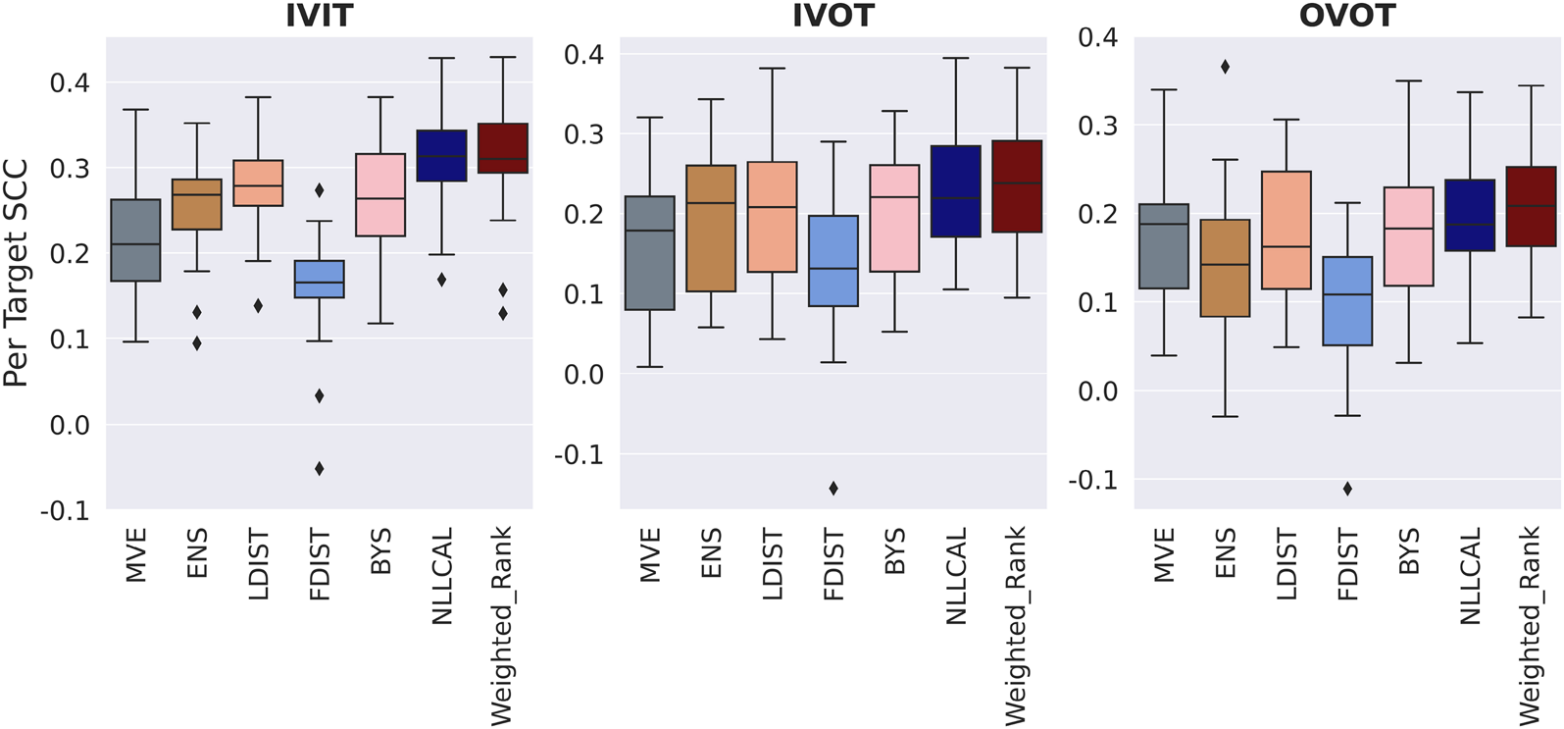
Boxplots reporting the SCCs of each model across all datasets. A higher SCC indicates stronger ranking ability

**Fig. 3:**
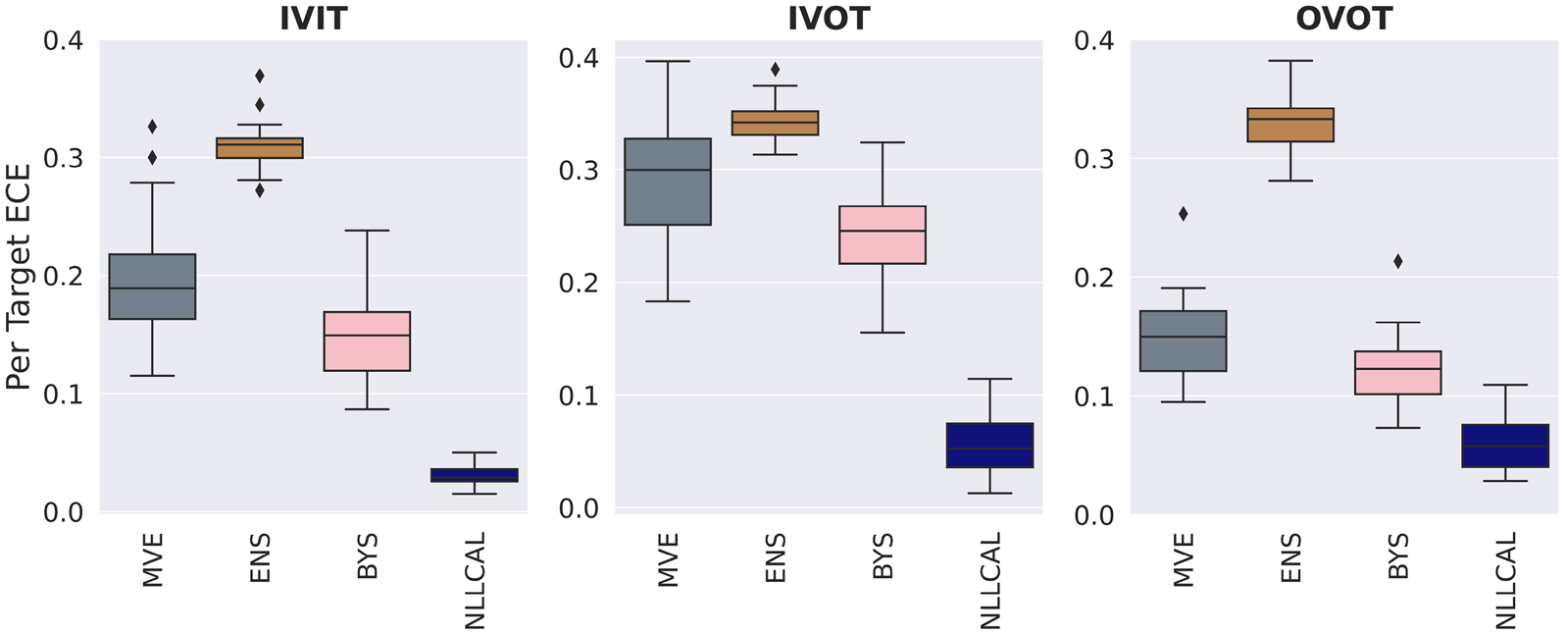
Boxplots reporting the ECEs of each model across all datasets. A lower ECE indicates stronger confidence-based calibration ability. The results of LDIST, FDIST and Weighted_Rank are not shown because the ECE metric is not applicable for these three methods

**Fig. 4:**
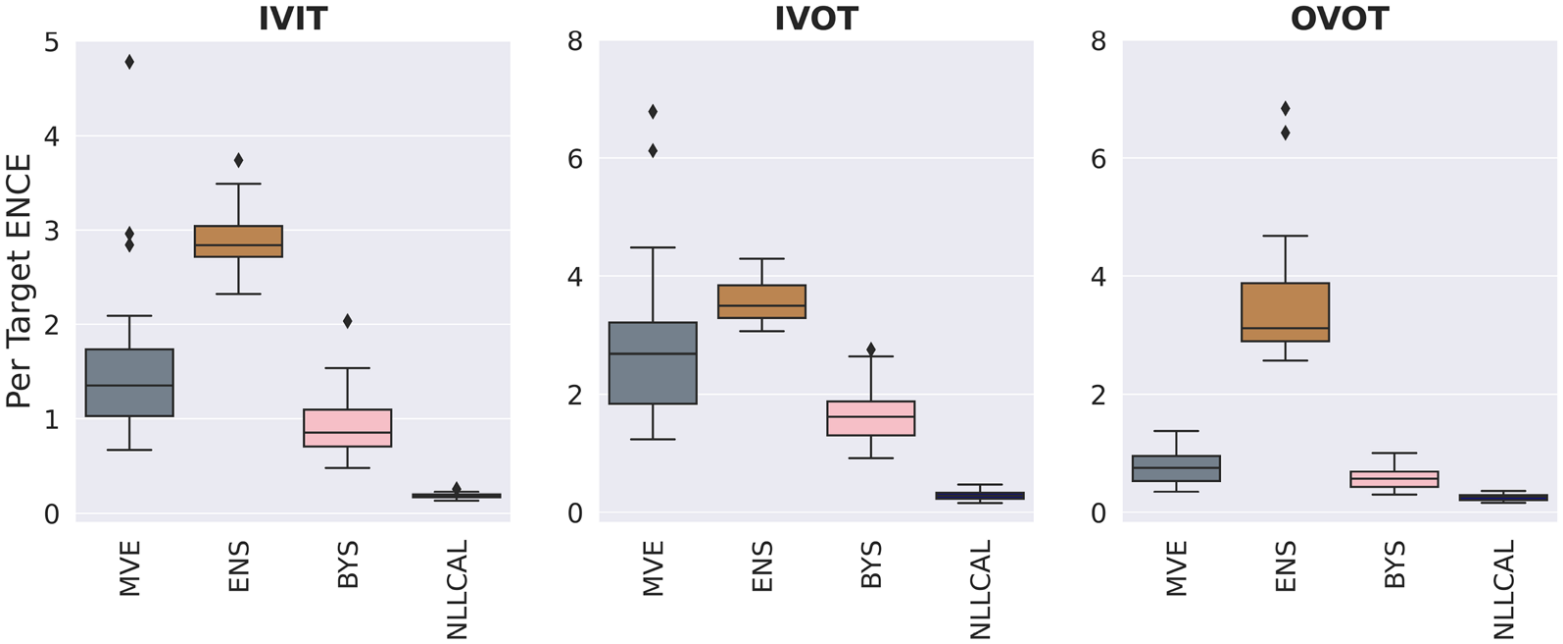
Boxplots reporting the ENCEs of each model across all datasets. A lower ENCE indicates stronger error-based calibration ability. The results of LDIST, FDIST and Weighted_Rank are not shown because the ENCE metric is not applicable for these three methods

The performance of four individual models (MVE, ENS, LDIST, FDIST) and NLLCAL across three data splitting strategies are provided in Table 1 by showing the values of SCC, ECE and ENCE. The SCCs of unweighted and weighted averaging models are shown in Table 2. The results of SCC, ECE and ENCE are also illustrated in Figs. 2, 3, 4 with box plots. For more detailed performance metrics on each target see Additional file 1: Tables S1–S9.

**Table 1:** Average performance of four individual models, BYS and NLLCAL

Metrics	Splitting strategies	MVE	ENS	LDIST	FDIST	BYS	NLLCAL
SCC	IVIT	0.212	0.257	0.281	0.161	0.263	**0.308**
	IVOT	0.154	0.193	0.202	0.134	0.198	**0.225**
	OVOT	0.171	0.142	0.174	0.101	0.183	**0.194**
ECE	IVIT	0.198	0.310	NA^*a*^	NA	0.151	**0.030**
	IVOT	0.295	0.344	NA	NA	0.243	**0.058**
	OVOT	0.149	0.332	NA	NA	0.121	**0.059**
ENCE	IVIT	1.576	2.903	NA	NA	0.943	**0.184**
	IVOT	2.849	3.582	NA	NA	1.656	**0.287**
	OVOT	0.773	3.575	NA	NA	0.572	**0.258**

Best results are highlighted in boldface type

A higher SCC, a lower ECE or a lower ENCE indicates better performance

^*a*^ECE and ENCE are not applicable for LDIST and FDIST

**Table 2:** Average performance (SCC) of weighted averaging models with different combinations of normalization functions and weighted methods

Splitting strategies	Weighted methods	MinMax	Zscore	Rank
IVIT	Unweighted	0.306	0.315	**0.314**
	Weighted	**0.314**	**0.317**	0.312
IVOT	Unweighted	0.230	0.236	**0.240**
	Weighted	**0.239**	**0.239**	0.236
OVOT	Unweighted	0.200	**0.207**	**0.211**
	Weighted	**0.204**	0.206	0.208

Better results between the weighted and unweighted approaches for each combination of splitting strategy and normalization function are highlighted in boldface type

A higher SCC indicates better performance

As expected, the performance of individual models for IVIT is generally better than that for IVOT and OVOT, since uncertainty estimation for in-domain samples is easier than that for out-of-domain samples. Among four individual models, LDIST always showed the best ranking ability and FDIST always showed the worst. According to the results of ECE and ENCE, MVE consistently showed a better performance compared with ENS at the task of confidence-based and error-based calibration.

We next examine the performance of BYS which combines ENS and MVE. For ranking ability, it is found that BYS achieved better or at least similar performance compared with the best performing individual model. A clearer trend is observed for calibration tasks where BYS consistently significantly surpassed MVE and ENS.

The results of NLLCAL presented in the last column of Table 1 clearly show that the performance of BYS can be further improved by performing post-hoc calibration on the validation set. In fact, NLLCAL outperformed all of the baseline models that were not calibrated including BYS, regardless of splitting strategies and evaluation metrics. A previous study suggested that the post-hoc calibration is expected to show good performance in independent and identically distributed regimes, but may fail in the conditions of distributional shift, even when the shift is minor [28]. Accordingly, it is not surprising that NLLCAL showed a satisfied performance for IVIT. However, as shown in Table 1, NLLCAL still achieved better SCC, ECE and ENCE compared with BYS for IVOT and OVOT, which suggests that it is worth calibrating the prediction on the validation set even in the out-of-domain scenario.

Different from NLLCAL, unweighted averaging strategy does not require the calibration process and therefore possesses wider application scenarios. Table 2 shows that with a proper normalization strategy, unweighted averaging models could achieve higher SCC compared with baseline models and even NLLCAL (0.314 VS 0.308 for IVIT, 0.240 VS 0.225 for IVOT and 0.211 VS 0.194 for OVOT). Considering the normalization strategy, there was no significant difference between MaxMin, Zscore and Rank, despite the observation that Rank seems to show a slightly higher performance.

If a weighted strategy was used for averaging models, the same situation with NLLCAL arose where calibration had a high probability of further improving the performance for Zscore-based and MinMax-based averaging models. For example, the mean SCC of MinMax-based averaging model improved from 0.306 to 0.314 for IVIT, from 0.230 to 0.239 for IVOT and from 0.200 to 0.204 for OVOT. However, it is observed that the SCCs of Rank-based averaging models decrease slightly with the weighted strategy.

**Fig. 5:**
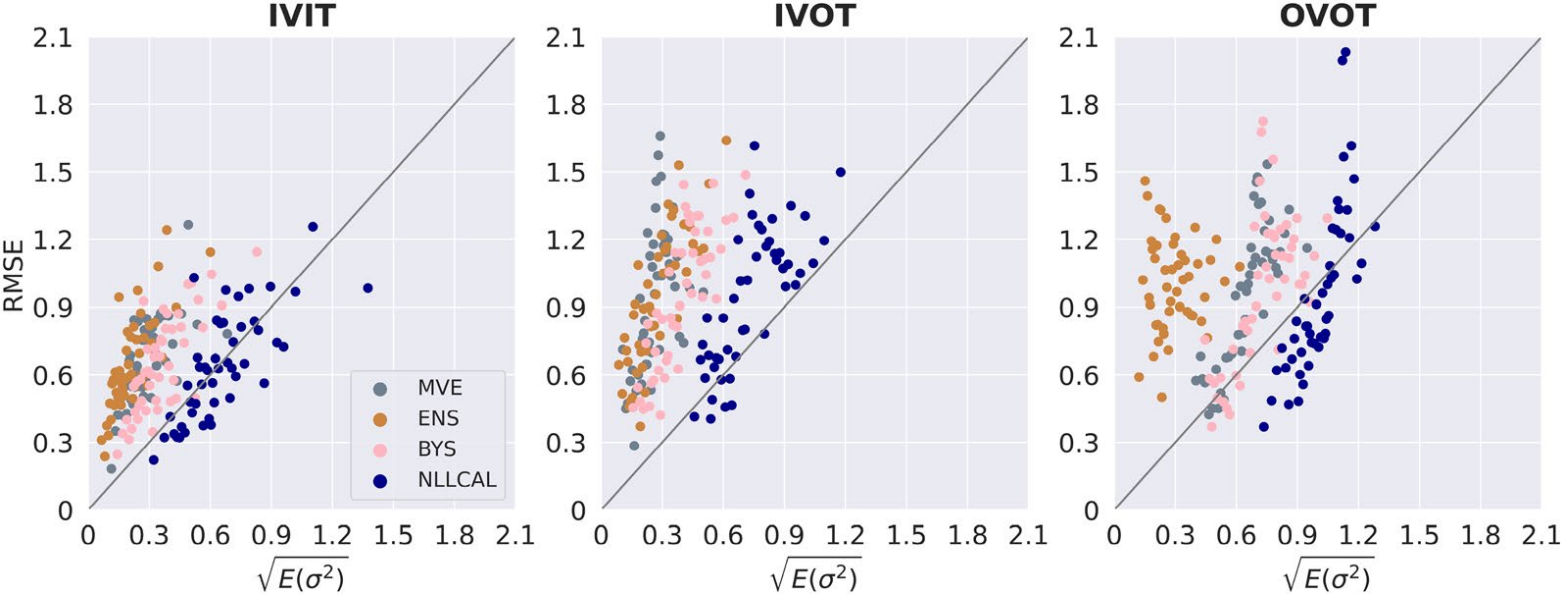
Error-based calibration plots for the first fold of erbB1. Each dot represents a bin containing 20 molecules. The *y*-axis indicates the RMSE for the bin and the *x*-axis indicates the root mean uncertainty estimated by the model. For an ideal model the dots should be distributed around the diagonal line

**Fig. 6:**
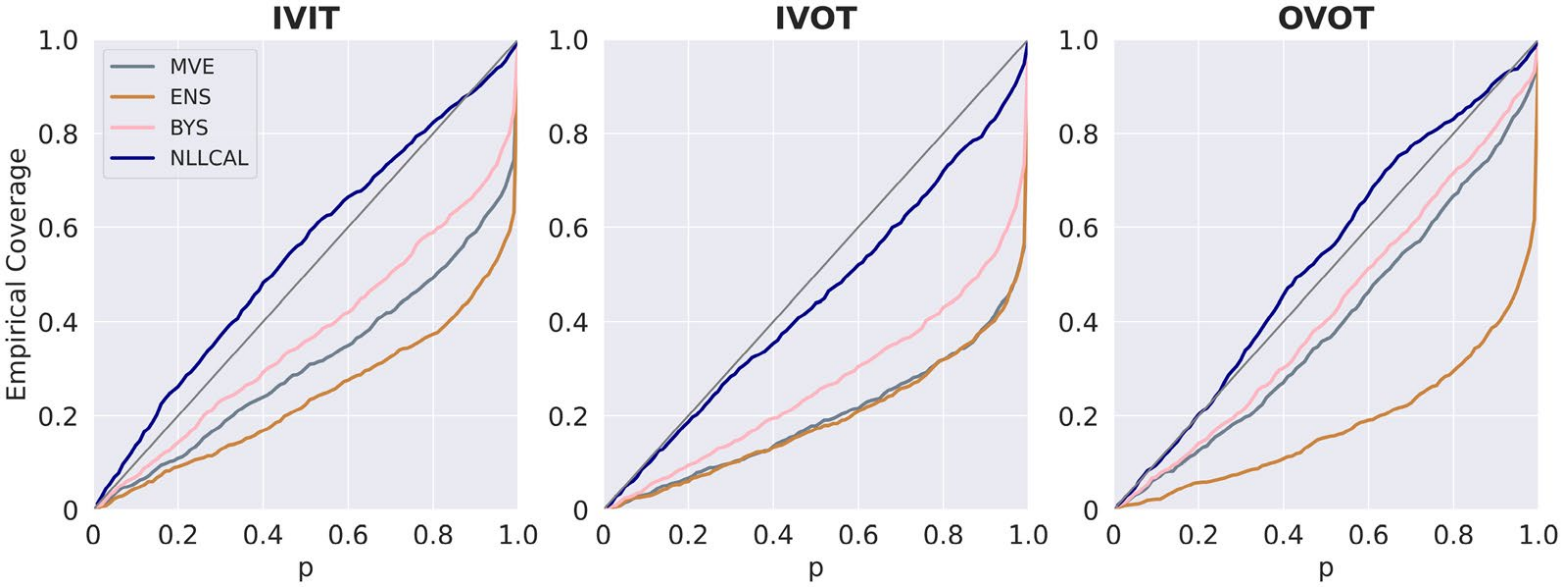
Confidence-based calibration plots for the first fold of erbB1. The *x*-axis indicates the confidence level varied from 0.0 to 1.0 and the corresponding observed probabilities that the labels fall into the estimated confidence intervals (Empirical Coverage) are shown along the *y*-axis. For an ideal model the curve should be around the diagonal line

**Fig. 7:**
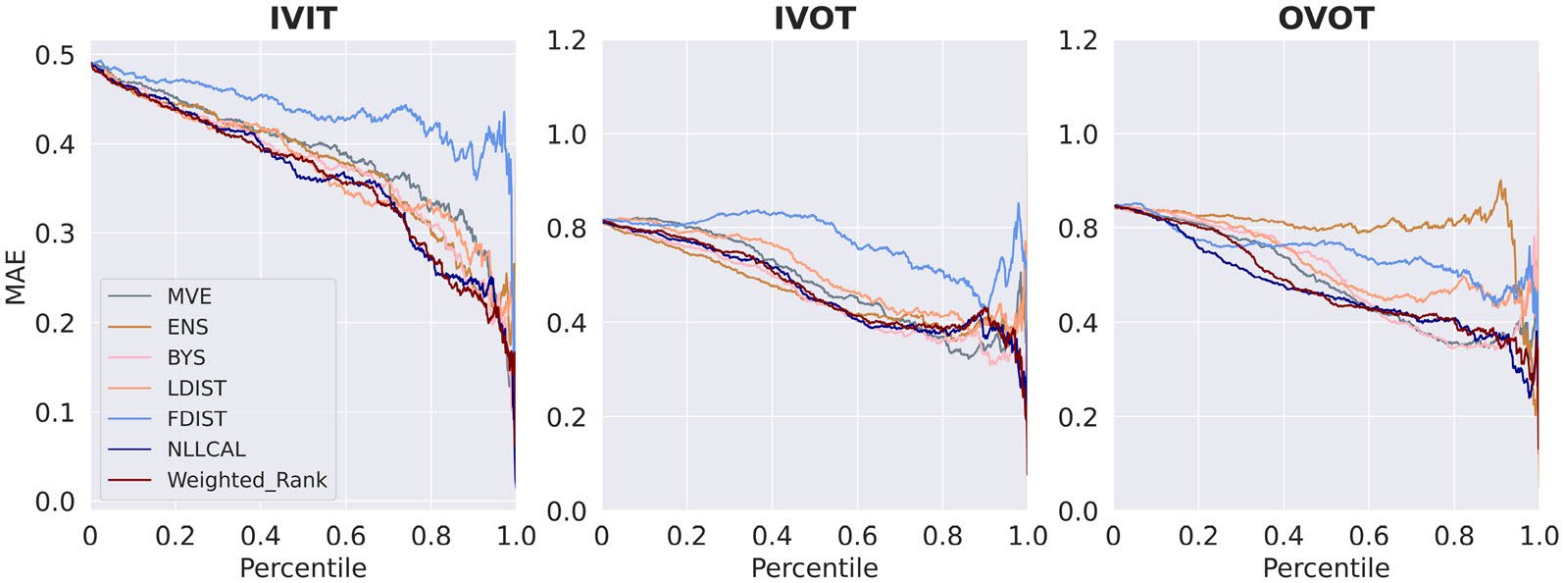
Confidence curves for the first fold of erbB1. This plot shows how the error (*y*-xis) on the subset varies if different proportions (*x*-axis) of molecules with the highest uncertainty are removed. An ideal model should present a monotonically decreasing curve

For visually presenting and comparing the results of different methods, the error-based calibration plots, confidence-based calibration plots and confidence curves for the first fold of erbB1 are shown in Figs. 5, 6, and 7, respectively. As one can observe in Figs. 5 and 6, NLLCAL shows a better calibration ability by performing post-hoc calibration on the validation set. The results of MVE and ENS were both significantly miscalibrated, especially for IVOT. Figure 5 also demonstrates that although in the IVOT setting the NLLCAL was still miscalibrated compared with the “ideal” diagonal line, it still outperformed BYS which indicates that post-hoc calibration is, to some extent, robust to the domain shift. Figure 7 demonstrates that for all splits weighted averaging method and NLLCAL could achieve better or comparable results with respect to the best performing individual model.

**Fig. 8:**
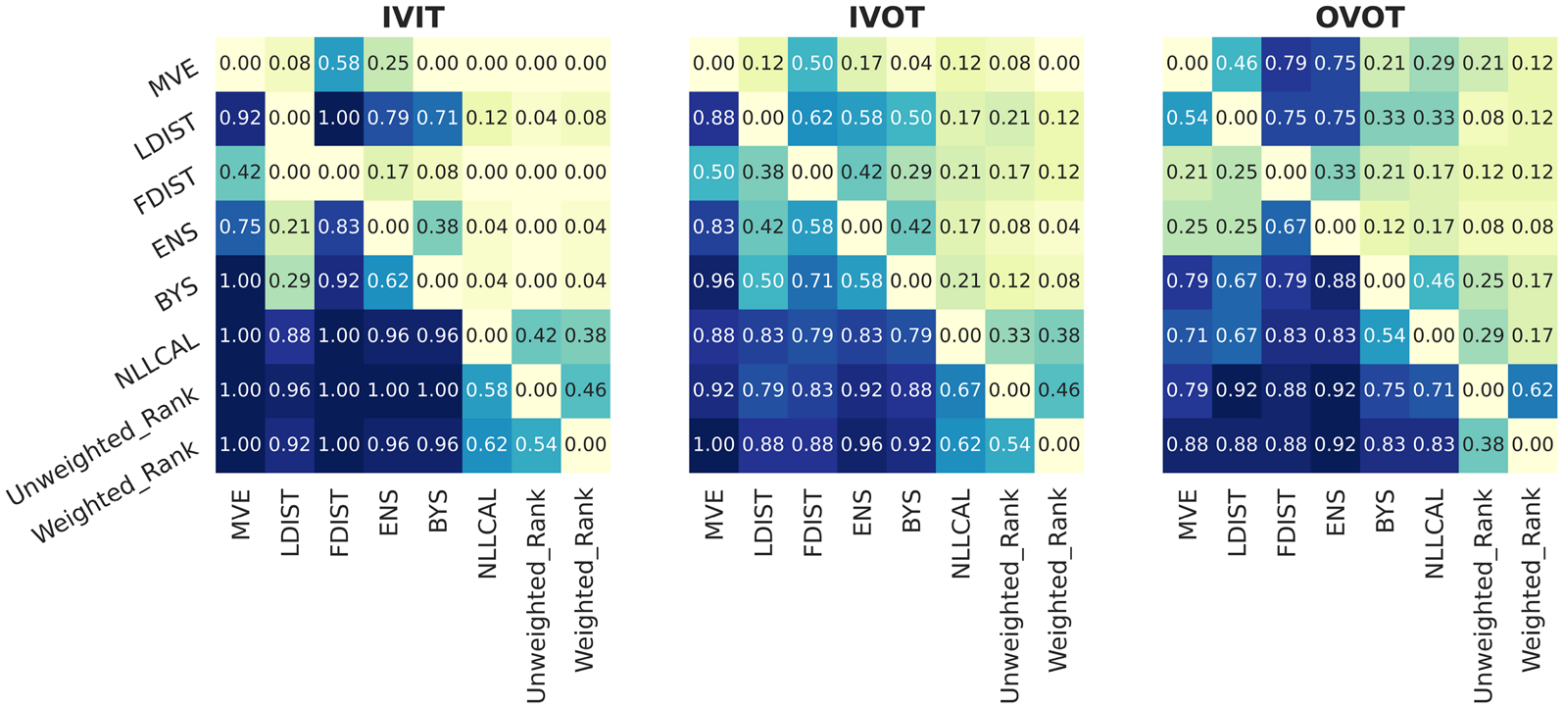
The frequency for a model (*y*-axis) to outperform another model (*x*-axis) across all targets with respect to the metric SCC. Since a model always performs equally with itself, the values on the diagonal lines are zeros. Rank is used as the representation of different normalizations

Except for the mean values, we also examined the relative performance of different methods across all 24 datasets. Taken one target as a single mission, Fig. 8 shows how often a model (*y*-axis) outperformed another (*x*-axis) which is defined by obtaining a higher mean value of SCC. As it can be seen, the improvement of using consensus strategy is outstanding and robust. For all targets and data splitting strategies, merely using Unweighted_Rank alone without performing post-hoc calibration is already very likely to get better performance compared with the traditional BYS method. This is especially ideal for real-world deployment.

### Ablation studies highlight the importance of each individual model

Ablation studies were performed to assess the effect of each individual model. First, for weighted averaging methods (using Weighted_Rank as the representative model), each single method was removed in turn during the construction of the consensus model and four new sub-models were built whose performance would be compared with that of the whole model. The results are shown in Table 3. As it can be seen, for IVIT and OVOT, the performance of the consensus model built using the entirely four types of uncertainties surpassed that of sub-models. For IVOT, the sub-model excluding MVE shows slightly better performance compared with the whole model. According to Eq. 2, MVE learns aleatoric uncertainty from the distribution of training set, thus it is reasonable to find that MVE did not perform well on out-of-domain test sets, as used in IVOT.

**Table 3:** Ablation study of different individual components of Weighted_Rank

Combinations	Splitting strategies		
	IVIT	IVOT	OVOT
E + L + F	0.306	**0.237**	0.195
M + L + F	0.302	0.228	0.198
M + E + F	0.296	0.229	0.197
M + E + L	0.300	0.216	0.196
M + E + F + L	**0.312**	0.236	**0.208**

SCC is used as the evaluation metric

Each letter represents an individual method: M refers to MVE, E refers to ENS, L refers to LDIST and F refers to FDIST

Best results are highlighted in boldface type for each splitting strategy

**Fig. 9:**
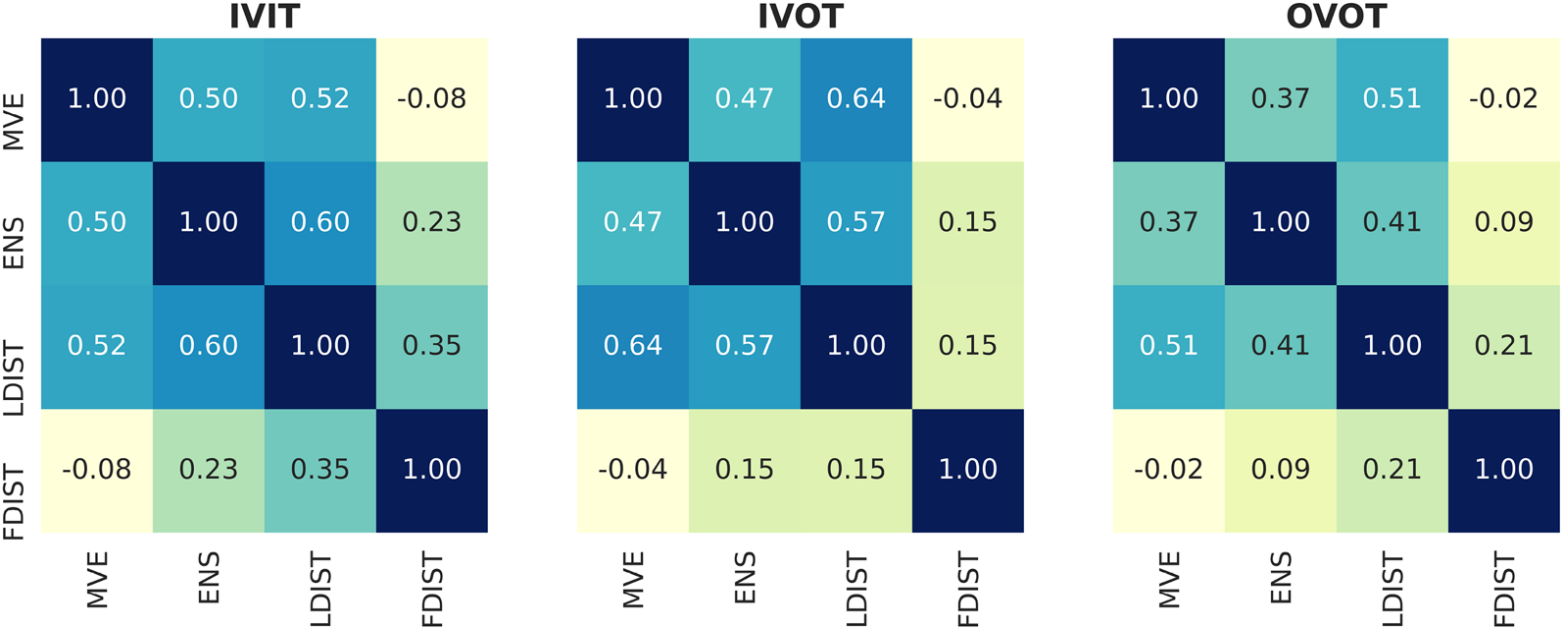
The correlation between the rank ordering of four individual models. Spearman rank correlation coefficients are annotated in the corresponding squares

**Fig. 10:**
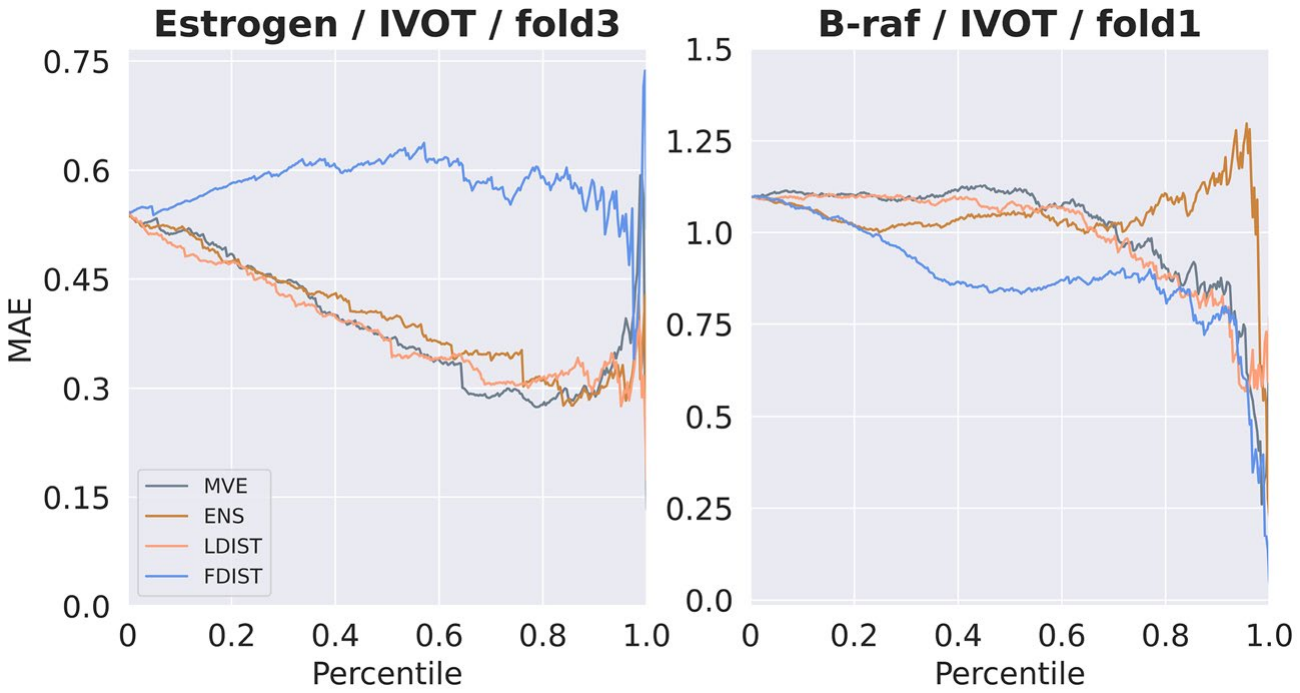
Confidence curves of two representative cases. (left) Estrogen, IVOT, fold3; (right) B-raf, IVOT, fold1

It is also interesting to note that although FDIST showed the worst ranking ability among the individual models (as shown in Table 1), the sub-model which excluded FDIST (M + E + L) showed significant decrease in SCC. We suppose that this is due to the strong complementary effect of FDIST. To prove this point, the correlation of predictions between the four individual models for each data splitting strategy are shown in Fig. 9. Correlation coefficients are calculated by averaging over different folds and targets. From a rank-order point of view, no matter for which data splitting method, there exists relatively high correlation between MVE, ENS, and LDIST. However, FDIST was weakly related with these three strategies, especially when IVOT and OVOT were adopted. In Fig. 10 we show two representative examples. In the left part of Fig. 10, all individual models except FDIST showed decreasing confidence curves, while FDIST failed to give a meaningful prediction. On the contrary, in the right part, the performance of other models was rather poor, but FDIST showed a satisfied decreasing curve.

Since FDIST is not related with the task or model, we assume that it can be treated as a prior estimation of the reliability. As in Bayesian inference, the nonconformity between the likelihood and the prior does not necessarily evidence that the prior is inappropriate, but may be due to the data bias or a mis-specified model [26]. FDIST could function as prior knowledge and would show its unique value in some cases, especially for a dataset with biased features, which can be utilized through the consensus strategy we proposed.

One more thing should be noticed in Fig. 9 is the high correlation between MVE and ENS. Technically, MVE and ENS capture the aleatoric and epistemic uncertainties, respectively, which are conceptually orthogonal, so it is expected that these two methods should be independent. However, the results shown in Fig. 9 suggest that these two methods are highly correlated on the whole. We suppose that it is because MVE, ENS and LDIST are all derived from the final layer of the neural network, but FDIST is calculated directly from the original representation of the molecule. The same phenomenon has also been reported by Scalia et al. [27].

As for NLLCAL, we compared the performance of three models: considering LDIST only (L), considering MVE and ENS together (M + E) and finally the fully constructed model (M + E + L + F). Only performing calibration using LDIST was previously proposed by Janet et al. [19] and thus taken as the baseline model for comparison. M + E was also taken into consideration in order to assess the value of incorporating chemical space distance into the consensus model. The results of ablation study are shown in Table 4. As it can be seen, M + E + L + F which used the whole four individual models showed the best performance for most metrics, except for ECE and ENCE in the OVOT setting that M + E performed equally or slightly better.

**Table 4:** Ablation study of individual components of NLLCAL

Metrics	Combinations	Splitting strategies		
		IVIT	IVOT	OVOT
SCC	L	0.281	0.202	0.174
	M + E	0.273	0.197	0.177
	M + E + L + F	**0.308**	**0.225**	**0.194**
ECE	L	0.035	0.065	0.062
	M + E	0.036	0.074	**0.059**
	M + E + L + F	**0.030**	**0.058**	**0.059**
ENCE	L	0.192	0.337	0.272
	M + E	0.188	0.377	**0.249**
	M + E + L + F	**0.184**	**0.287**	0.258

Each letter represents an individual method: M refers to MVE, E refers to ENS, L refers to LDIST and F refers to FDIST

Best results are highlighted in boldface type for each splitting strategy

The results discussed above reveal that all four individual models have its unique value no matter for weighted averaging or NLLCAL. The total consensus model considering all of the four individual models showed the best performance.

### Comparison of NLLCAL with conformal prediction

As mentioned in the “Methods and datasets” section, a confidence-based calibrated uncertainty method, like NLLCAL, can be used to make interval estimation according to Eq. 1. It is noticed that another widely used approach for generating the prediction interval is the Conformal Prediction (CP) [54]. In this case, we compared the performance of these two methods in the mission of prediction interval estimation. For detailed explanation of Conformal Prediction methodology we refer the readers to Ref [54], and for different ways of defining nonconformity values, to Ref [55]. We adopted the following ESD (ensemble standard deviation) nonconformity function [55] to calculate the nonconformity value:

22 $$\alpha_{i} = \frac{{\left| {y_{i} - \hat{y}_{i} } \right|}}{{e^{{\sqrt {Q_{E} \left( {x_{i} } \right)} }} }}$$

where $Q_{E} \left( {x_{i} } \right)$ is the estimation obtained from the ENS approach as shown in Eq. 5, $y_{i}$ is the label and $\hat{y}_{i}$ is the predicted value. The confidence level was set to 0.9 for all experiments. Validity and efficiency were used to evaluate the performance of confidence interval predictors. Validity of the predictor was assessed by calculating the average fraction of labels falling inside the prediction interval across all folds for a single target. This value is expected to be as close to 0.9, the confidence level we set, as possible. Efficiency of the predictor was calculated as the average range of the prediction interval generated. For example, for an interval of 5.74 ± 0.38, the efficiency is 0.76. Provided the validity is close to 0.9, the efficiency is the lower the better. We say a model is valid for a target if the validity value falls into [0.85, 0.95].

The results of validity and efficiency of NLLCAL and CP are shown in Additional file 1: Tables S10, S11, respectively. For IVIT, both NLLCAL and CP were valid for all 24 targets, while NLLCAL could on average generate narrower intervals than CP (2.147 VS 2.205). For OVOT, NLLCAL and CP showed comparable performance that both of them were valid on 17 targets. Although the test set used in OVOT is out-of-domain, NLLCAL and CP were still able to generate valid estimation for most targets owing to the similar residual distribution between the validation set and the test set. However, for IVOT, it can be observed that CP was valid for only two targets (Carbonic and COX-1). The validity values of CP for the rest targets are all much lower than the confidence level we set, indicating that the prediction intervals given by CP were generally too narrow. It is not surprising since calibration on an in-domain validation set will definitely lead to underestimation of residuals on an out-of-domain test set. In fact, strictly speaking, CP is not applicable in the condition of IVOT and OVOT since the use of CP requires the randomness assumption that samples are independently drawn from the same distribution [54]. For the same reason, the prediction intervals given by NLLCAL were also generally too narrow for IVOT, but NLLCAL was still able to give valid estimation for 7 targets and showed better average validity value compared with CP (0.833 VS 0.782). All of these results clearly show that NLLCAL can generate valid prediction intervals with practical usefulness in the IVIT setting, while at the same time show more robust performance in domain shift settings compared with the Conformal Prediction approach.

### Mean–variance estimation does not decrease model performance

One thing remains unclear is that whether the pipeline we proposed would affect the model performance, since we hope that any additional strategies for better uncertainty quantification will not decrease the accuracy of the origin model, which is the most important thing we concern about. Obviously, FDIST and LDIST have no influence on the prediction process. ENS requires to ensemble several individually trained models, which has been widely used to improve the robustness of the model. However, whether using mean–variance estimation would affect model accuracy still remains to be unknown. To investigate this effect, we retrained all models with the normal MSE loss, whose results were taken as the baseline for comparison. The fivefold CV RMSEs on each target are reported in Additional file 1: Table S12 for both MSE loss and mean–variance loss (referred to as MVE in the table). As it can be seen, mean–variance loss achieved lower RMSE values compared with that obtained by MSE loss for most cases. The mean RMSE over 24 datasets got a minor improvement by using mean–variance loss for all three splitting strategies.

The results reported in Additional file 1: Table S12 indicate that mean–variance loss may have the added advantage of further driving down the prediction error on the test set. Similar phenomena have also been reported by some other researches [9, 27, 48]. With mean–variance loss, MVE is able to capture the aleatoric uncertainty [variance of the conditional distribution of target variables $p(y{|}x)$] under the heteroskedastic assumption. Considering that heteroskedasticity is very common in bioactivity datasets, mean–variance loss can be treated as a useful regularization technique for avoiding overfitting on high noise samples, which may explain the improvement for model accuracy. However, whether this kind of regularization effect can be generalized to other datasets and tasks still needs to be further studied under more systematic tests, which is beyond the scope of this study.

### Efficient uncertainty quantification for machine learning models is necessary and needs to be further explored

Although this research focuses on constructing hybrid uncertainty quantification methods for deep learning models as stated in the title, we wondered whether the similar consensus approach could also work for machine learning models. Random forest (RF) was taken as the representative model for studies. All experiments were reperformed by replacing the D-MPNN model with the RF model trained using the Scikit-learn package [56]. The default parameter values were used during training, where the number of trees was set to 100. It is reported that more trees generally do not lead to improved performance for QSAR regression modeling [57]. ECFP4 fingerprint was used to featurize molecules. Since LDIST and MVE are not applicable for RF models, we only attempted to build the hybrid models by combining ENS and FDIST together. ENS was calculated using the variance of predictions generated by 100 trees.

The performance of RF models across all targets are presented in Additional file 1: Table S13 by showing the average RMSE values. Additional file 1: Table S14 reports the performance of ENS, FDIST, Weighted_Rank and NLLCAL in the same way of Table 1 by showing the average SCC, ECE and ENCE. As it can be seen, ENS showed much better ranking ability than that of FDIST and performed well regarding to the calibration tasks. However, by combining these two individual models using weighted averaging and post-hoc calibration, Weighted_Rank and NLLCAL still showed comparable or better performance compared with ENS.

Although GCNN models, like D-MPNN we studied in this research, have gained great attention in QSAR modeling and shown outstanding performance on large datasets, machine learning models still have unique value that should not be omitted. Generally speaking, machine learning models are much faster for training (Additional file 1: Figure S1) and more convenient for parameter tunning and interpretation. Jiang et al. even proposed that descriptor-based machine learning models on average showed better performance on small datasets than GCNN models [58]. As Additional file 1: Table S14 shows, the hybrid algorithm may also be beneficial for uncertainty quantification of machine learning models. However, how to choose individual methods and make combination is still an open question that needs to be further studied. In conclusion, efficient uncertainty quantification for deep learning and traditional machine learning models are equally important in QSAR modeling. We will continue to explore these two aspects in the future studies.

## Conclusion

Data-driven methods are emerging as important tools for drug design and discovery. To fully realize the potential of these models, well-calibrated uncertainty quantification can be as important as accurate predictions. Many uncertainty quantification strategies have been proposed and benchmarked in the context of deep-learning-based QSAR regression modeling in recent studies. However, it has been reported that these approaches have the deficiency of showing large performance variation across different datasets and model architectures. In this study we explored several consensus strategies for improving the performance of the individual model. We found that both weighted averaging and post-hoc calibration on the validation set could lead to better performance. The importance of incorporating chemical space distance information into traditional Bayesian framework is also highlighted. Although the performance improvement is promising, there still exists gap between the reliability of the model and the need for real-world deployment. Considering the consensus strategies used in this study are rather simple, future work could focus on the transformation of chemical space distance information into the prior distribution that can be effectively used by existing Bayesian uncertainty quantification approaches.

## Supplementary Information

**Additional file 1: Table S1–S9.** Detailed fivefold CV performance metrics (SCC, ECE and ENCE) on each target for four individual models (MVE, ENS, LDIST, FDIST), NLLCAL, Weighted_Rank and Unweigthed_Rank. **Table S10–S11.** Fivefold CV validity and efficiency values of NLLCAL and CP for prediction interval estimation. **Table S12.** Fivefold CV RMSEs of D-MPNN models trained with different loss functions on each target. **Table S13.** Fivefold CV RMSEs of RF models on each target. **Table S14.** Average performance of ENS, FDIST, Weighted_Rank and NLLCAL for RF models. **Figure S1.** Summary of the average training time for RF models and D-MPNN models on each target in the IVIT setting.

## Data Availability

The datasets, codes and files used in this study are available at https://github.com/wangdingyan/HybridUQ. Python 3.7.0 was used as the programming language. The training of D-MPNN models was based on the Chemprop package (https://github.com/chemprop/chemprop). Python libraries used in the study include numpy (1.16.5, https://github.com/numpy/numpy), pandas (1.2.3, https://github.com/pandas-dev/pandas), scipy (1.4.1, https://github.com/scipy/scipy), scikit-learn (https://github.com/scikit-learn/scikit-learn), pytorch (1.4.0, https://github.com/pytorch/pytorch), tqdm (4.51.0, https://github.com/tqdm/tqdm), typed-argument-parser (1.7.0, https://github.com/swansonk14/typed-argument-parser), and rdkit (1.9.0, https://github.com/rdkit/rdkit).

## Abbreviations

QSAR: Quantitative Structure-Activity Relationship
AD: Applicability Domain
GCNN: Graph Convolutional Neural Network
D-MPNN: Directed Message Passing Neural Network
MVE: Mean-Variance Estimation
MLE: Maximum Likelihood Estimation
MSE: Mean Squared Error
ENS: Ensemble
FDIST: Feature Space Distance
MHFP6: MinHash Fingerprint6
LDIST: Latent Space Distance
BYS: Bayesian Approach
NLL: Negative Log-Likelihood
SCC: Spearman Correlation Coefficient
MAE: Mean Absolute Error
NLLCAL: Negative Log-Likelihood Calibration
AUC: Area Under the Curve
RMSE: Root Mean Squared Error
ENCE: Expected Normalized Calibration Error
ECE: Expected Calibration Error
CV: Cross Validation
RF: Random Forest
CP: Conformal Prediction
ESD: Ensemble Standard Deviation

## References

[1] Muratov EN, Bajorath J, Sheridan RP, Tetko IV, Filimonov D, Poroikov V, Oprea TI, Baskin II, Varnek A, Roitberg A (2020). QSAR without borders. Chem Soc Rev.

[2] Jiménez-Luna J, Grisoni F, Schneider G (2020). Drug discovery with explainable artificial intelligence. Nat Mach Intell.

[3] Mervin LH, Johansson S, Semenova E, Giblin KA, Engkvist O (2021). Uncertainty quantification in drug design. Drug Discov Today.

[4] Nigam A, Pollice R, Hurley MFD, Hickman RJ, Aldeghi M, Yoshikawa N, Chithrananda S, Voelz VA, Aspuru-Guzik A (2021). Assigning confidence to molecular property prediction. Expert Opin Drug Discov.

[5] Hie B, Bryson BD, Berger B (2020). Leveraging uncertainty in machine learning accelerates biological discovery and design. Cell Syst.

[6] Begoli E, Bhattacharya T, Kusnezov D (2019). The need for uncertainty quantification in machine-assisted medical decision making. Nat Mach Intell.

[7] Zhang Y, Lee AA (2019). Bayesian semi-supervised learning for uncertainty-calibrated prediction of molecular properties and active learning. Chem Sci.

[8] Rakhimbekova A, Madzhidov TI, Nugmanov RI, Gimadiev TR, Baskin II, Varnek A (2020). Comprehensive analysis of applicability domains of QSPR models for chemical reactions. Int J Mol Sci.

[9] Goodall REA, Lee AA (2020). Predicting materials properties without crystal structure: deep representation learning from stoichiometry. Nat Commun.

[10] Jonas E, Kuhn S (2019). Rapid prediction of NMR spectral properties with quantified uncertainty. J Cheminform.

[11] Wen MJ, Tadmor EB (2020). Uncertainty quantification in molecular simulations with dropout neural network potentials. Npj Comput Mater.

[12] Sheridan RP, Feuston BP, Maiorov VN, Kearsley SK (2004). Similarity to molecules in the training set is a good discriminator for prediction accuracy in QSAR. J Chem Inf Comput Sci.

[13] Toplak M, Mocnik R, Polajnar M, Bosnic Z, Carlsson L, Hasselgren C, Demsar J, Boyer S, Zupan B, Stalring J (2014). Assessment of machine learning reliability methods for quantifying the applicability domain of QSAR regression models. J Chem Inf Model.

[14] Sheridan RP (2015). The relative importance of domain applicability metrics for estimating prediction errors in QSAR varies with training set diversity. J Chem Inf Model.

[15] Liu R, Glover KP, Feasel MG, Wallqvist A (2018). General approach to estimate error bars for quantitative structure-activity relationship predictions of molecular activity. J Chem Inf Model.

[16] Berenger F, Yamanishi Y (2019). A distance-based boolean applicability domain for classification of high throughput screening data. J Chem Inf Model.

[17] Liu R, Wallqvist A (2019). Molecular similarity-based domain applicability metric efficiently identifies out-of-domain compounds. J Chem Inf Model.

[18] Tagasovska N, Lopez-Paz D (2018) Single-model uncertainties for deep learning. https://arxiv.org/abs/1811.00908

[19] Janet JP, Duan C, Yang T, Nandy A, Kulik HJ (2019). A quantitative uncertainty metric controls error in neural network-driven chemical discovery. Chem Sci.

[20] Kendall A, Gal Y (2017) What uncertainties do we need in Bayesian deep learning for computer vision? arXiv e-prints. https://arxiv.org/abs/1703.04977

[21] Gal Y, Ghahramani Z (2015) Dropout as a Bayesian approximation: representing model uncertainty in deep learning. https://arxiv.org/abs/1506.02142

[22] Amini A, Schwarting W, Soleimany A, Rus D (2019) Deep evidential regression. arXiv e-prints. https://arxiv.org/abs/1910.02600

[23] Sun S, Chen C, Carin L (2017) Learning structured weight uncertainty in bayesian neural networks. In: Aarti S, Jerry Z (eds) proceedings of the 20th international conference on artificial intelligence and statistics. Vol 54. Proceedings of machine learning research: PMLR; p 1283–1292

[24] Ryu S, Kwon Y, Kim WY (2019). A Bayesian graph convolutional network for reliable prediction of molecular properties with uncertainty quantification. Chem Sci.

[25] Beker W, Wolos A, Szymkuc S, Grzybowski BA (2020). Minimal-uncertainty prediction of general drug-likeness based on Bayesian neural networks. Nat Mach Intell.

[26] van de Schoot R, Depaoli S, King R, Kramer B, Märtens K, Tadesse MG, Vannucci M, Gelman A, Veen D, Willemsen J (2021). Bayesian statistics and modelling. Nat Rev Methods Primers.

[27] Scalia G, Grambow CA, Pernici B, Li YP, Green WH (2020). Evaluating scalable uncertainty estimation methods for deep learning-based molecular property prediction. J Chem Inf Model.

[28] Ovadia Y, Fertig E, Ren J, Nado Z, Sculley D, Nowozin S, Dillon JV, Lakshminarayanan B, Snoek J (2019) Can you trust your model’s uncertainty? Evaluating predictive uncertainty under dataset shift. arXiv e-prints. https://arxiv.org/abs/1906.02530

[29] Malinin A, Gales M (2018) Predictive uncertainty estimation via prior networks. Advances in neural information processing systems 31 (Nips 2018) https://arxiv.org/abs/1802.10501

[30] Charpentier B, Zügner D, Günnemann S (2020) Posterior network: uncertainty estimation without OOD samples via density-based pseudo-counts. Curran Associates, Inc: New York, NY

[31] Tetko IV, Sushko I, Pandey AK, Zhu H, Tropsha A, Papa E, Oberg T, Todeschini R, Fourches D, Varnek A (2008). Critical assessment of QSAR models of environmental toxicity against *Tetrahymena pyriformis*: focusing on applicability domain and overfitting by variable selection. J Chem Inf Model.

[32] Cortes-Ciriano I, Firth NC, Bender A, Watson O (2018). Discovering highly potent molecules from an initial set of inactives using iterative screening. J Chem Inf Model.

[33] Mendez D, Gaulton A, Bento AP, Chambers J, De Veij M, Felix E, Magarinos MP, Mosquera JF, Mutowo P, Nowotka M (2019). ChEMBL: towards direct deposition of bioassay data. Nucleic Acids Res.

[34] Cortes-Ciriano I, Bender A (2019). Deep confidence: a computationally efficient framework for calculating reliable prediction errors for deep neural networks. J Chem Inf Model.

[35] Cortes-Ciriano I, Bender A (2019). Reliable prediction errors for deep neural networks using test-time dropout. J Chem Inf Model.

[36] Watson OP, Cortes-Ciriano I, Taylor AR, Watson JA (2019). A decision-theoretic approach to the evaluation of machine learning algorithms in computational drug discovery. Bioinformatics.

[37] Yang K, Swanson K, Jin W, Coley C, Eiden P, Gao H, Guzman-Perez A, Hopper T, Kelley B, Mathea M (2019). Analyzing learned molecular representations for property prediction. J Chem Inf Model.

[38] Vaswani A, Shazeer N, Parmar N, Uszkoreit J, Jones L, Gomez AN, Kaiser L, Polosukhin I (2017) Attention is all you need. arXiv e-prints. https://arxiv.org/abs/1706.03762

[39] Lakshminarayanan B, Pritzel A, Blundell C (2016) Simple and scalable predictive uncertainty estimation using deep ensembles. https://arxiv.org/abs/1612.01474

[40] Gal Y, Hron J, Kendall A (2017) Concrete dropout. https://arxiv.org/abs/1705.07832

[41] Wenzel F, Snoek J, Tran D, Jenatton R (2020) Hyperparameter ensembles for robustness and uncertainty quantification. arXiv e-prints. https://arxiv.org/abs/2006.13570

[42] Peterson AA, Christensen R, Khorshidi A (2017). Addressing uncertainty in atomistic machine learning. Phys Chem Chem Phys.

[43] Ashukha A, Lyzhov A, Molchanov D, Vetrov D (2020) Pitfalls of in-domain uncertainty estimation and ensembling in deep learning. arXiv e-prints. https://arxiv.org/abs/2002.06470

[44] Sahigara F, Mansouri K, Ballabio D, Mauri A, Consonni V, Todeschini R (2012). Comparison of different approaches to define the applicability domain of QSAR models. Molecules.

[45] Probst D, Reymond JL (2018). A probabilistic molecular fingerprint for big data settings. J Cheminform.

[46] Rogers D, Hahn M (2010). Extended-connectivity fingerprints. J Chem Inf Model.

[47] Nikolova N, Jaworska J (2004). Approaches to measure chemical similarity—a review. Qsar Comb Sci.

[48] Hirschfeld L, Swanson K, Yang K, Barzilay R, Coley CW (2020). Uncertainty quantification using neural networks for molecular property prediction. J Chem Inf Model.

[49] Sheridan RP (2012). Three useful dimensions for domain applicability in QSAR models using random forest. J Chem Inf Model.

[50] Sheridan RP (2013). Using random forest to model the domain applicability of another random forest model. J Chem Inf Model.

[51] Levi D, Gispan L, Giladi N, Fetaya E (2019) Evaluating and calibrating uncertainty prediction in regression tasks. arXiv e-prints. https://arxiv.org/abs/1905.11659 10.3390/s22155540 PMC9330317 35898047

[52] Li X, Li Z, Wu X, Xiong Z, Yang T, Fu Z, Liu X, Tan X, Zhong F, Wan X (2020). Deep learning enhancing kinome-wide polypharmacology profiling: model construction and experiment validation. J Med Chem.

[53] Mysinger MM, Carchia M, Irwin JJ, Shoichet BK (2012). Directory of useful decoys, enhanced (DUD-E): better ligands and decoys for better benchmarking. J Med Chem.

[54] Norinder U, Carlsson L, Boyer S, Eklund M (2014). Introducing conformal prediction in predictive modeling. A transparent and flexible alternative to applicability domain determination. J Chem Inf Model.

[55] Svensson F, Aniceto N, Norinder U, Cortes-Ciriano I, Spjuth O, Carlsson L, Bender A (2018). Conformal regression for quantitative structure-activity relationship modeling-quantifying prediction uncertainty. J Chem Inf Model.

[56] Pedregosa F, Varoquaux G, Gramfort A, Michel V, Thirion B, Grisel O, Blondel M, Prettenhofer P, Weiss R, Dubourg V (2011). Scikit-learn: machine learning in python. J Mach Learn Res.

[57] Cortes-Ciriano I, van Westen GJP, Bouvier G, Nilges M, Overington JP, Bender A, Malliavin TE (2016). Improved large-scale prediction of growth inhibition patterns using the NCI60 cancer cell line panel. Bioinformatics.

[58] Jiang DJ, Wu ZX, Hsieh CY, Chen GY, Liao B, Wang Z, Shen C, Cao DS, Wu JA, Hou TJ (2021). Could graph neural networks learn better molecular representation for drug discovery? A comparison study of descriptor-based and graph-based models. J Cheminform.

