# Supplementary material for: A hybrid framework for improving uncertainty quantification in deep learning-based QSAR regression modeling
Source: J Cheminform. 2021 Sep 20;13:69. doi: 10.1186/s13321-021-00551-x (PMC8454160; doi:10.1186/s13321-021-00551-x)
Supplement: Supplementary file 1 — Additional file 1: Table S1–S9. Detailed fivefold CV performance metrics (SCC, ECE and ENCE) on each target for four individual models (MVE, ENS, LDIST, FDIST), NLLCAL, Weighted_Rank and Unweigthed_Rank. Table S10–S11. Fivefold CV validity and efficiency values of NLLCAL and CP for prediction interval estimation. Table S12. Fivefold CV RMSEs of D-MPNN models trained with different loss functions on each target. Table S13. Fivefold CV RMSEs of RF models on each target. Table S14. Average performance of ENS, FDIST, Weighted_Rank and NLLCAL for RF models. Figure S1. Summary of the average training time for RF models and D-MPNN models on each target in the IVIT setting. [file 13321_2021_551_MOESM1_ESM.pdf]

Additional file 1

A Hybrid Framework for Improving Uncertainty Quantification in Deep Learning-Based QSAR Regression Modeling

Dingyan Wang,[a,b,c,‡] Jie Yu,[b,c,‡] Lifan Chen,[b,c] Xutong Li,[b,c] Hualiang Jiang,[b,c] Kaixian Chen,[b,c] Mingyue Zheng,[b,c,*] Xiaomin Luo[a,b,c,*]

[a] Shanghai Key Laboratory of Forensic Medicine, Academy of Forensic Science, Shanghai 200063, China.

[b] University of Chinese Academy of Sciences, No.19A Yuquan Road, Beijing 100049, China.

[c] Drug Discovery and Design Center, State Key Laboratory of Drug Research, Shanghai Institute of Materia Medica, Chinese Academy of Sciences, 555 Zuchongzhi Road, Shanghai 201203, China.

Table S1. 5-fold CV SCCs of different models on each dataset in the IVIT setting

Targets	MVE	ENS	BYS	FDIST	LDIST	Unweighted_Rank	Weighted_Rank	NLLCAL
A2a	0.098	0.094	0.118	0.097	0.248	0.189	0.157	0.198
ABL1	0.096	0.131	0.134	0.115	0.139	0.178	0.129	0.169
Acetylcholinesterase	0.219	0.350	0.326	0.177	0.317	0.382	0.380	0.366
Aurora-A	0.205	0.253	0.256	0.148	0.253	0.298	0.294	0.289
B-raf	0.112	0.266	0.218	0.190	0.282	0.299	0.311	0.315
Cannabinoid	0.177	0.232	0.243	0.183	0.253	0.291	0.293	0.295
Carbonic	0.333	0.282	0.376	0.182	0.382	0.387	0.391	0.403
Caspase	0.345	0.325	0.363	-0.052	0.358	0.391	0.392	0.378
Coagulation	0.188	0.228	0.231	0.235	0.270	0.299	0.296	0.301
COX-1	0.368	0.352	0.382	0.273	0.362	0.434	0.429	0.428
COX-2	0.296	0.286	0.321	0.146	0.339	0.352	0.354	0.359
Dihydrofolate	0.169	0.217	0.202	0.220	0.261	0.293	0.299	0.312
Dopamine	0.215	0.278	0.271	0.219	0.308	0.348	0.350	0.342
Ephrin	0.160	0.217	0.221	0.193	0.255	0.278	0.280	0.269
erbB1	0.259	0.272	0.299	0.116	0.285	0.316	0.318	0.317
Estrogen	0.286	0.266	0.315	0.033	0.275	0.330	0.337	0.333
Glucocorticoid	0.125	0.225	0.188	0.184	0.268	0.277	0.276	0.262
Glycogen	0.274	0.326	0.330	0.161	0.293	0.355	0.349	0.335
HERG	0.217	0.287	0.280	0.162	0.285	0.316	0.310	0.318
JAK2	0.218	0.270	0.278	0.157	0.284	0.308	0.310	0.294
LCK	0.189	0.233	0.239	0.237	0.256	0.309	0.309	0.304
Monoamine	0.240	0.310	0.299	0.169	0.310	0.359	0.357	0.347
opioid	0.188	0.286	0.255	0.154	0.275	0.318	0.316	0.240
Vanilloid	0.119	0.179	0.164	0.158	0.191	0.220	0.238	0.209
Mean	0.212	0.257	0.263	0.161	0.281	0.314	0.312	0.308

Table S2. 5-fold CV SCCs of different models on each dataset in the IVOT setting

Targets	MVE	ENS	BYS	FDIST	LDIST	Unweighted_Rank	Weighted_Rank	NLLCAL
A2a	0.244	0.313	0.258	0.125	0.306	0.362	0.252	0.206
ABL1	0.008	0.066	0.052	0.162	0.105	0.135	0.141	0.137
Acetylcholinesterase	0.186	0.269	0.263	0.204	0.208	0.295	0.291	0.291
Aurora-A	0.099	0.104	0.107	0.195	0.121	0.192	0.178	0.174
B-raf	0.031	0.065	0.080	0.209	0.110	0.152	0.137	0.140
Cannabinoid	0.046	0.187	0.137	0.206	0.129	0.198	0.211	0.196
Carbonic	0.249	0.328	0.316	0.290	0.382	0.374	0.380	0.395
Caspase	0.320	0.311	0.328	-0.143	0.328	0.310	0.336	0.292
Coagulation	0.080	0.063	0.086	0.063	0.042	0.088	0.095	0.105
COX-1	0.268	0.257	0.290	0.085	0.262	0.300	0.304	0.298
COX-2	0.184	0.218	0.232	0.081	0.219	0.240	0.241	0.237
Dihydrofolate	0.224	0.057	0.217	0.123	0.212	0.210	0.225	0.172
Dopamine	0.157	0.343	0.287	0.279	0.267	0.357	0.357	0.282
Ephrin	0.191	0.236	0.260	0.160	0.208	0.278	0.275	0.281
erbB1	0.188	0.232	0.246	0.014	0.196	0.228	0.234	0.232
Estrogen	0.182	0.210	0.223	0.016	0.301	0.290	0.291	0.293
Glucocorticoid	0.087	0.152	0.148	0.180	0.155	0.202	0.190	0.192
Glycogen	0.128	0.142	0.150	0.126	0.135	0.198	0.188	0.169
HERG	0.175	0.216	0.217	0.137	0.213	0.250	0.247	0.254
JAK2	0.079	0.140	0.134	0.167	0.151	0.185	0.174	0.185
LCK	0.066	0.098	0.096	0.118	0.096	0.138	0.130	0.113
Monoamine	0.221	0.234	0.255	0.117	0.264	0.284	0.287	0.269
opioid	0.222	0.321	0.284	0.232	0.336	0.381	0.382	0.374
Vanilloid	0.063	0.070	0.078	0.077	0.115	0.114	0.124	0.116
Mean	0.154	0.193	0.198	0.134	0.202	0.240	0.236	0.225

Table S3. 5-fold CV SCCs of different models on each dataset in the OVOT setting

Targets	MVE	ENS	BYS	FDIST	LDIST	Unweighted_Rank	Weighted_Rank	NLLCAL
A2a	0.244	0.242	0.266	0.137	0.285	0.320	0.270	0.247
ABL1	0.039	-0.029	0.031	0.212	0.083	0.103	0.130	0.126
Acetylcholinesterase	0.196	0.173	0.210	0.145	0.188	0.247	0.242	0.226
Aurora-A	0.115	0.141	0.139	0.179	0.119	0.197	0.190	0.165
B-raf	0.123	0.070	0.111	0.095	0.119	0.145	0.150	0.145
Cannabinoid	0.083	0.079	0.117	0.178	0.144	0.172	0.164	0.178
Carbonic	0.269	0.231	0.286	0.212	0.272	0.298	0.306	0.282
Caspase	0.339	0.366	0.350	-0.111	0.306	0.338	0.344	0.337
Coagulation	0.115	0.079	0.118	-0.029	0.048	0.068	0.097	0.094
COX-1	0.174	0.085	0.183	0.047	0.140	0.171	0.182	0.177
COX-2	0.203	0.148	0.211	0.031	0.180	0.196	0.215	0.201
Dihydrofolate	0.210	0.035	0.164	0.077	0.100	0.121	0.197	0.190
Dopamine	0.179	0.113	0.182	0.152	0.297	0.282	0.251	0.268
Ephrin	0.210	0.204	0.233	0.106	0.244	0.258	0.255	0.234
erbB1	0.255	0.175	0.260	0.052	0.220	0.259	0.274	0.282
Estrogen	0.206	0.145	0.203	0.006	0.256	0.255	0.226	0.210
Glucocorticoid	0.058	0.143	0.103	0.125	0.088	0.159	0.157	0.154
Glycogen	0.122	0.133	0.167	0.100	0.144	0.192	0.187	0.177
HERG	0.210	0.192	0.219	0.102	0.195	0.244	0.238	0.184
JAK2	0.175	0.125	0.174	0.150	0.128	0.219	0.202	0.159
LCK	0.057	-0.004	0.064	0.134	0.078	0.118	0.093	0.112
Monoamine	0.201	0.196	0.228	0.110	0.212	0.260	0.236	0.209
opioid	0.227	0.261	0.261	0.192	0.257	0.323	0.305	0.252
Vanilloid	0.087	0.112	0.115	0.023	0.072	0.116	0.082	0.053
Mean	0.171	0.142	0.183	0.101	0.174	0.211	0.208	0.194

Table S4. 5-fold CV ECEs of different models on each dataset in the IVIT setting.

Targets	MVE	ENS	BYS	NLLCAL
A2a	0.192	0.369	0.166	0.050
ABL1	0.115	0.316	0.087	0.023
Acetylcholinesterase	0.326	0.295	0.238	0.048
Aurora-A	0.208	0.296	0.154	0.026
B-raf	0.236	0.315	0.180	0.026
Cannabinoid	0.173	0.281	0.128	0.037
Carbonic	0.143	0.303	0.110	0.041
Caspase	0.165	0.288	0.117	0.030
Coagulation	0.212	0.303	0.161	0.026
COX-1	0.147	0.317	0.114	0.036
COX-2	0.182	0.298	0.137	0.032
Dihydrofolate	0.190	0.315	0.152	0.032
Dopamine	0.137	0.345	0.113	0.031
Ephrin	0.213	0.313	0.165	0.015
erbB1	0.188	0.272	0.132	0.036
Estrogen	0.140	0.300	0.107	0.026
Glucocorticoid	0.185	0.328	0.148	0.020
Glycogen	0.278	0.313	0.216	0.026
HERG	0.300	0.302	0.225	0.029
JAK2	0.268	0.306	0.204	0.028
LCK	0.158	0.311	0.120	0.023
Monoamine	0.232	0.320	0.178	0.027
opioid	0.190	0.320	0.151	0.040
Vanilloid	0.173	0.311	0.134	0.018
Mean	0.198	0.310	0.151	0.030

Table S5. 5-fold CV ECEs of different models on each dataset in the IVOT setting

Targets	MVE	ENS	BYS	NLLCAL
A2a	0.278	0.389	0.251	0.115
ABL1	0.183	0.355	0.156	0.088
Acetylcholinesterase	0.396	0.349	0.314	0.037
Aurora-A	0.245	0.345	0.207	0.049
B-raf	0.378	0.374	0.324	0.113
Cannabinoid	0.283	0.330	0.230	0.094
Carbonic	0.235	0.313	0.186	0.045
Caspase	0.302	0.331	0.242	0.074
Coagulation	0.297	0.332	0.238	0.041
COX-1	0.225	0.340	0.186	0.036
COX-2	0.311	0.327	0.249	0.019
Dihydrofolate	0.252	0.362	0.221	0.075
Dopamine	0.293	0.375	0.262	0.076
Ephrin	0.323	0.347	0.264	0.036
erbB1	0.313	0.329	0.252	0.067
Estrogen	0.248	0.332	0.203	0.035
Glucocorticoid	0.348	0.362	0.293	0.066
Glycogen	0.302	0.320	0.242	0.035
HERG	0.375	0.321	0.283	0.013
JAK2	0.355	0.331	0.285	0.068
LCK	0.225	0.344	0.190	0.069
Monoamine	0.321	0.340	0.264	0.043
opioid	0.259	0.351	0.220	0.056
Vanilloid	0.339	0.344	0.277	0.035
Mean	0.295	0.344	0.243	0.058

Table S6. 5-fold CV ECEs of different models on each dataset in the OVOT setting

Targets	MVE	ENS	BYS	NLLCAL
A2a	0.126	0.363	0.122	0.091
ABL1	0.095	0.325	0.081	0.078
Acetylcholinesterase	0.187	0.312	0.146	0.029
Aurora-A	0.171	0.315	0.136	0.050
B-raf	0.253	0.339	0.213	0.095
Cannabinoid	0.114	0.334	0.088	0.068
Carbonic	0.097	0.281	0.073	0.054
Caspase	0.134	0.311	0.103	0.063
Coagulation	0.151	0.354	0.126	0.054
COX-1	0.096	0.349	0.079	0.043
COX-2	0.137	0.340	0.111	0.033
Dihydrofolate	0.185	0.379	0.162	0.078
Dopamine	0.109	0.382	0.096	0.109
Ephrin	0.166	0.311	0.128	0.033
erbB1	0.156	0.334	0.124	0.028
Estrogen	0.149	0.308	0.118	0.029
Glucocorticoid	0.122	0.315	0.104	0.093
Glycogen	0.173	0.332	0.135	0.034
HERG	0.161	0.310	0.124	0.042
JAK2	0.186	0.315	0.146	0.057
LCK	0.126	0.339	0.103	0.075
Monoamine	0.190	0.338	0.154	0.059
opioid	0.169	0.355	0.142	0.060
Vanilloid	0.118	0.325	0.090	0.065
Mean	0.149	0.332	0.121	0.059

Table S7. 5-fold CV ENCEs of different models on each dataset in the IVIT setting

Targets	MVE	ENS	BYS	NLLCAL
A2a	1.093	3.357	0.851	0.184
ABL1	0.668	2.927	0.478	0.165
Acetylcholinesterase	4.780	3.036	2.035	0.190
Aurora-A	1.602	2.733	0.957	0.171
B-raf	1.907	2.955	1.144	0.221
Cannabinoid	1.387	2.570	0.862	0.205
Carbonic	0.954	3.058	0.667	0.198
Caspase	1.099	2.718	0.706	0.201
Coagulation	1.575	2.810	0.987	0.175
COX-1	0.991	3.199	0.723	0.227
COX-2	1.288	2.781	0.852	0.178
Dihydrofolate	1.721	3.487	1.118	0.256
Dopamine	0.908	3.737	0.633	0.151
Ephrin	1.334	2.526	0.823	0.148
erbB1	1.409	2.319	0.817	0.177
Estrogen	0.933	2.825	0.646	0.170
Glucocorticoid	1.197	2.956	0.797	0.133
Glycogen	2.960	2.962	1.500	0.189
HERG	2.843	2.776	1.538	0.175
JAK2	2.089	2.611	1.202	0.183
LCK	0.910	2.649	0.620	0.158
Monoamine	1.775	2.854	1.092	0.199
opioid	1.374	3.107	0.874	0.194
Vanilloid	1.039	2.715	0.701	0.160
Mean	1.576	2.903	0.943	0.184

Table S8. 5-fold CV ENCEs of different models on each dataset in the IVOT setting

Targets	MVE	ENS	BYS	NLLCAL
A2a	1.803	4.075	1.419	0.369
ABL1	1.394	3.814	0.913	0.387
Acetylcholinesterase	6.788	3.650	2.754	0.274
Aurora-A	1.525	3.475	1.096	0.219
B-raf	4.220	4.185	2.416	0.468
Cannabinoid	2.463	3.193	1.566	0.404
Carbonic	1.920	3.065	1.234	0.181
Caspase	2.660	3.562	1.625	0.403
Coagulation	2.733	3.289	1.569	0.230
COX-1	1.429	3.434	1.034	0.242
COX-2	2.906	3.323	1.735	0.176
Dihydrofolate	1.851	4.295	1.321	0.302
Dopamine	2.318	4.232	1.701	0.271
Ephrin	2.926	3.318	1.679	0.199
erbB1	2.700	3.071	1.598	0.324
Estrogen	1.620	3.288	1.134	0.255
Glucocorticoid	3.900	4.142	2.237	0.310
Glycogen	2.776	3.133	1.639	0.226
HERG	6.126	3.554	2.639	0.284
JAK2	4.485	3.365	2.229	0.335
LCK	1.231	3.514	0.923	0.297
Monoamine	3.429	3.917	2.116	0.327
opioid	2.025	3.795	1.366	0.265
Vanilloid	3.140	3.268	1.802	0.157
Mean	2.849	3.582	1.656	0.287

Table S9. 5-fold CV ENCEs of different models on each dataset in the OVOT setting

Targets	MVE	ENS	BYS	NLLCAL
A2a	0.491	4.588	0.416	0.316
ABL1	0.395	3.528	0.324	0.278
Acetylcholinesterase	1.040	2.856	0.739	0.208
Aurora-A	0.976	2.720	0.683	0.267
B-raf	1.359	3.927	1.001	0.338
Cannabinoid	0.434	3.067	0.300	0.263
Carbonic	0.702	2.774	0.484	0.270
Caspase	0.862	3.057	0.635	0.323
Coagulation	0.706	4.683	0.560	0.287
COX-1	0.456	3.653	0.371	0.198
COX-2	0.749	3.857	0.543	0.196
Dihydrofolate	0.964	6.431	0.721	0.340
Dopamine	0.346	6.842	0.297	0.296
Ephrin	0.822	2.569	0.575	0.177
erbB1	0.696	3.003	0.533	0.190
Estrogen	0.750	2.865	0.576	0.199
Glucocorticoid	0.540	2.906	0.434	0.360
Glycogen	0.903	2.911	0.608	0.159
HERG	1.068	3.163	0.800	0.265
JAK2	0.934	2.944	0.673	0.259
LCK	0.519	3.973	0.431	0.259
Monoamine	1.372	3.205	0.908	0.274
opioid	0.946	3.412	0.691	0.238
Vanilloid	0.531	2.858	0.402	0.233
Mean	0.773	3.575	0.572	0.258

Table S10. 5-fold CV validity values of NLLCAL and CP for prediction interval estimation on each target [a]

Targets	IVIT		IVOT		OVOT	
	NLLCAL	CP	NLLCAL	CP	NLLCAL	CP
A2a	0.879	0.904	0.804	0.794	0.799	0.820
ABL1	0.897	0.909	0.784	0.761	0.851	0.863
Acetylcholinesterase	0.905	0.898	0.845	0.777	0.890	0.894
Aurora-A	0.901	0.901	0.845	0.772	0.858	0.860
B-raf	0.902	0.902	0.772	0.597	0.817	0.824
Cannabinoid	0.880	0.893	0.784	0.744	0.853	0.883
Carbonic	0.875	0.902	0.866	0.851	0.859	0.905
Caspase	0.898	0.895	0.813	0.779	0.864	0.843
Coagulation	0.896	0.907	0.837	0.759	0.819	0.826
COX-1	0.909	0.896	0.874	0.858	0.886	0.899
COX-2	0.898	0.892	0.880	0.835	0.870	0.867
Dihydrofolate	0.887	0.879	0.843	0.787	0.811	0.828
Dopamine	0.866	0.901	0.804	0.782	0.823	0.818
Ephrin	0.898	0.892	0.880	0.830	0.900	0.895
erbB1	0.899	0.897	0.815	0.770	0.902	0.907
Estrogen	0.908	0.898	0.858	0.802	0.885	0.888
Glucocorticoid	0.893	0.892	0.808	0.714	0.845	0.859
Glycogen	0.896	0.881	0.847	0.834	0.878	0.881
HERG	0.903	0.898	0.889	0.834	0.911	0.896
JAK2	0.906	0.895	0.798	0.727	0.876	0.884
LCK	0.886	0.895	0.800	0.750	0.814	0.846
Monoamine	0.898	0.898	0.839	0.808	0.883	0.895
opioid	0.881	0.897	0.828	0.789	0.877	0.877
Vanilloid	0.889	0.890	0.884	0.812	0.885	0.871
Mean	0.894	0.896	0.833	0.782	0.861	0.868

[a] Values falling outside [0.85, 0.95] are underlined.

Table S11. 5-fold CV efficiency values of NLLCAL and CP for prediction interval estimation on each target

Targets	IVIT		IVOT		OVOT	
	NLLCAL	CP	NLLCAL	CP	NLLCAL	CP
A2a	2.282	2.532	2.894	2.751	3.006	3.257
ABL1	2.605	2.705	2.568	2.460	3.366	3.542
Acetylcholinesterase	2.142	2.125	2.665	2.208	3.069	3.110
Aurora-A	2.300	2.371	2.931	2.522	2.943	3.065
B-raf	1.801	1.834	2.747	1.924	3.392	3.431
Cannabinoid	2.166	2.238	2.658	2.401	3.161	3.392
Carbonic	1.717	1.899	1.895	1.785	2.067	2.361
Caspase	1.767	1.823	2.014	1.829	2.507	2.484
Coagulation	2.532	2.620	3.084	2.557	3.405	3.512
COX-1	2.060	2.084	2.256	2.157	2.365	2.501
COX-2	2.297	2.319	2.889	2.536	3.011	2.995
Dihydrofolate	2.502	2.526	3.273	2.899	3.394	3.331
Dopamine	2.441	2.678	3.015	2.748	2.935	3.052
Ephrin	2.000	1.988	2.451	2.134	2.591	2.519
erbB1	2.168	2.166	2.447	2.206	3.222	3.372
Estrogen	2.000	2.015	2.250	1.917	2.511	2.628
Glucocorticoid	1.851	1.835	2.385	1.937	2.711	2.792
Glycogen	2.325	2.310	2.662	2.539	3.166	3.211
HERG	1.729	1.697	2.006	1.722	2.319	2.165
JAK2	1.965	1.938	2.466	2.075	3.154	3.244
LCK	2.509	2.672	3.123	2.634	3.410	3.689
Monoamine	1.984	2.004	2.169	1.980	2.720	2.828
opioid	2.120	2.221	2.539	2.299	3.096	3.070
Vanilloid	2.268	2.318	2.837	2.328	2.742	2.735
Mean	2.147	2.205	2.593	2.273	2.928	3.012

Table S12. 5-fold CV RMSEs of D-MPNN models trained with different loss functions on each target

Targets	IVIT		IVOT		OVOT	
	MVE	MSE	MVE	MSE	MVE	MSE
A2a	0.790	0.823	1.118	1.182	1.162	1.210
ABL1	0.849	0.871	1.073	1.090	1.142	1.180
Acetylcholinesterase	0.693	0.708	0.974	0.993	0.976	1.000
Aurora-A	0.761	0.773	1.044	1.050	1.023	1.029
B-raf	0.599	0.612	1.148	1.206	1.212	1.287
Cannabinoid	0.747	0.764	1.103	1.083	1.108	1.114
Carbonic	0.603	0.610	0.680	0.699	0.733	0.770
Caspase	0.611	0.620	0.811	0.820	0.902	0.943
Coagulation	0.827	0.840	1.107	1.121	1.227	1.221
COX-1	0.673	0.679	0.774	0.775	0.780	0.776
COX-2	0.755	0.764	0.961	0.965	1.030	1.050
Dihydrofolate	0.846	0.860	1.138	1.106	1.206	1.195
Dopamine	0.840	0.852	1.129	1.146	1.044	1.052
Ephrin	0.653	0.654	0.805	0.804	0.809	0.810
erbB1	0.690	0.696	0.985	0.988	1.006	1.002
Estrogen	0.653	0.662	0.812	0.814	0.829	0.855
Glucocorticoid	0.600	0.608	0.930	0.944	0.892	0.904
Glycogen	0.752	0.761	0.928	0.962	1.009	1.030
HERG	0.561	0.562	0.738	0.739	0.762	0.765
JAK2	0.633	0.641	1.008	1.051	1.009	0.998
LCK	0.823	0.833	1.179	1.179	1.228	1.254
Monoamine	0.659	0.672	0.836	0.841	0.875	0.883
opioid	0.719	0.739	1.009	1.027	1.005	0.996
Vanilloid	0.726	0.725	0.889	0.889	0.862	0.868
Mean	0.711	0.722	0.966	0.978	0.993	1.008

Table S13. 5-fold CV RMSEs of RF models on each target

Targets	IVIT	IVOT	OVOT
A2a	0.763	0.962	0.995
ABL1	0.817	1.032	1.111
Acetylcholinesterase	0.779	1.035	1.037
Aurora-A	0.790	0.994	1.004
B-raf	0.630	1.035	1.243
Cannabinoid	0.750	1.045	1.104
Carbonic	0.608	0.785	0.795
Caspase	0.599	0.871	0.895
Coagulation	0.841	1.114	1.158
COX-1	0.701	0.814	0.810
COX-2	0.792	1.067	1.051
Dihydrofolate	0.854	1.048	1.102
Dopamine	0.756	0.950	1.101
Ephrin	0.678	0.825	0.848
erbB1	0.722	1.015	1.031
Estrogen	0.692	0.920	0.914
Glucocorticoid	0.596	0.950	0.924
Glycogen	0.733	0.945	0.955
HERG	0.622	0.790	0.809
JAK2	0.657	0.956	0.939
LCK	0.820	1.111	1.141
Monoamine	0.700	0.882	0.917
opioid	0.761	1.036	0.998
Vanilloid	0.736	0.890	0.882
Mean	0.725	0.961	0.990

Table S14. Average performance of ENS, FDIST, Weighted_Rank and NLLCAL for RF models [a]

Metrics	Splitting Strategies	ENS	FDIST	Weighted_Rank	NLLCAL
SCC	IVIT	0.325	0.191	0.325	**0.329**
	IVOT	0.248	0.127	**0.254**	**0.254**
	OVOT	0.229	0.124	0.231	**0.237**
ECE	IVIT	**0.025**	*NA* [b]	*NA*	0.033
	IVOT	0.050	*NA*	*NA*	**0.048**
	OVOT	**0.058**	*NA*	*NA*	**0.058**
ENCE	IVIT	0.211	*NA*	*NA*	**0.179**
	IVOT	0.248	*NA*	*NA*	**0.226**
	OVOT	0.285	*NA*	*NA*	**0.231**

[a] Best results are highlighted in boldface type.

[b] Not Applicable.

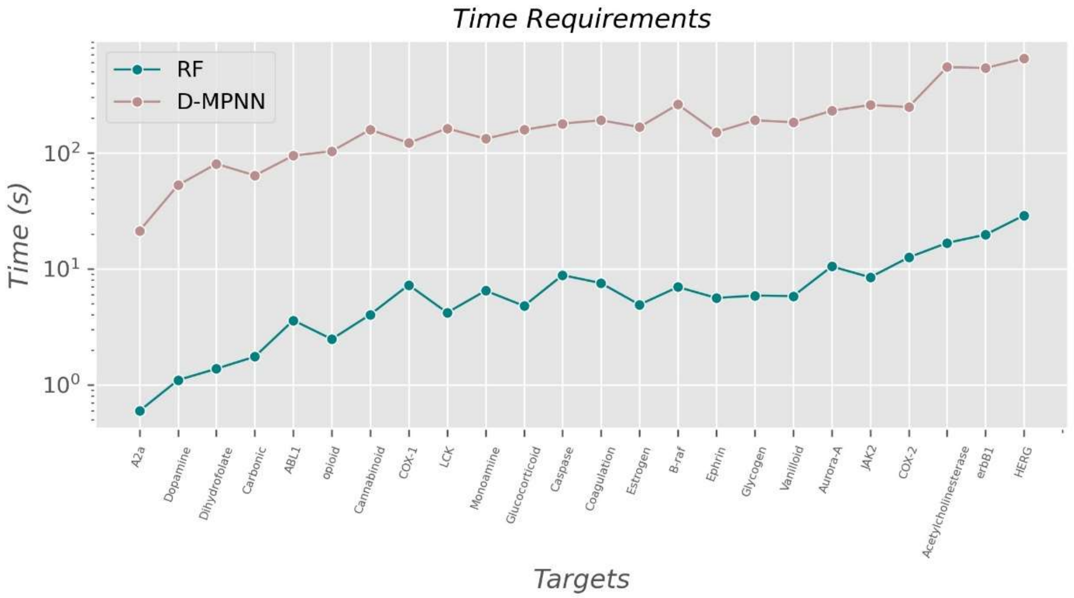

Figure S1. Summary of the average training time for RF models and D-MPNN models on each target in the IVIT setting. D-MPNN models were trained with one NVIDIA Tesla GPU with 16GB of memory.
